# Integrating central nervous system metagenomics and host response for diagnosis of tuberculosis meningitis and its mimics

**DOI:** 10.1038/s41467-022-29353-x

**Published:** 2022-03-30

**Authors:** P. S. Ramachandran, A. Ramesh, F. V. Cresswell, A. Wapniarski, R. Narendra, C. M. Quinn, E. B. Tran, M. K. Rutakingirwa, A. S. Bangdiwala, E. Kagimu, K. T. Kandole, K. C. Zorn, L. Tugume, J. Kasibante, K. Ssebambulidde, M. Okirwoth, N. C. Bahr, A. Musubire, C. P. Skipper, C. Fouassier, A. Lyden, P. Serpa, G. Castaneda, S. Caldera, V. Ahyong, J. L. DeRisi, C. Langelier, E. D. Crawford, D. R. Boulware, D. B. Meya, M. R. Wilson

**Affiliations:** 1 Weill Institute for Neurosciences, Department of Neurology, University of California, San Francisco, San Francisco, CA USA; 2 University of Melbourne, Melbourne, VIC Australia; 3 UCSF Center for Tuberculosis, San Francisco, CA USA; 4 UCSF Center for Encephalitis and Meningitis, San Francisco, CA USA; 5 Clinical Research Department, London School of Hygiene and Tropical Medicine, London, UK; 6 Infectious Diseases Institute, Makerere University, Kampala, Uganda; 7 Medical Research Council—Uganda Virus Research Institute—LSHTM Uganda Research Unit, Entebbe, Uganda; 8 University of California School of Medicine, San Francisco, CA USA; 9 University of Minnesota, Minneapolis, MN USA; 10 Department of Biochemistry and Biophysics, University of California, San Francisco, San Francisco, CA USA; 11 Division of Infectious Diseases, Department of Medicine, University of Kansas, Kansas City, KS USA; 12 Chan Zuckerberg Biohub, San Francisco, CA USA; 13 Department of Medicine, University of California, San Francisco, San Francisco, CA USA

**Keywords:** Infectious-disease diagnostics, Pathogens, Meningitis, Tuberculosis

## Abstract

The epidemiology of infectious causes of meningitis in sub-Saharan Africa is not well understood, and a common cause of meningitis in this region, *Mycobacterium tuberculosis* (TB), is notoriously hard to diagnose. Here we show that integrating cerebrospinal fluid (CSF) metagenomic next-generation sequencing (mNGS) with a host gene expression-based machine learning classifier (MLC) enhances diagnostic accuracy for TB meningitis (TBM) and its mimics. 368 HIV-infected Ugandan adults with subacute meningitis were prospectively enrolled. Total RNA and DNA CSF mNGS libraries were sequenced to identify meningitis pathogens. In parallel, a CSF host transcriptomic MLC to distinguish between TBM and other infections was trained and then evaluated in a blinded fashion on an independent dataset. mNGS identifies an array of infectious TBM mimics (and co-infections), including emerging, treatable, and vaccine-preventable pathogens including Wesselsbron virus, *Toxoplasma gondii, Streptococcus pneumoniae*, *Nocardia brasiliensis*, measles virus and cytomegalovirus. By leveraging the specificity of mNGS and the sensitivity of an MLC created from CSF host transcriptomes, the combined assay has high sensitivity (88.9%) and specificity (86.7%) for the detection of TBM and its many mimics. Furthermore, we achieve comparable combined assay performance at sequencing depths more amenable to performing diagnostic mNGS in low resource settings.

## Abstract (web summary)

Tuberculous meningitis is difficult to differentiate from meningitis caused by other pathogens. Here, the authors combine metagenomics-based pathogen detection in cerebrospinal fluid with a host gene expression-based machine learning classifier for diagnosis.

## Introduction

*Mycobacterium tuberculosis* (TB) affects 10 million (8.9–11 million) people worldwide and carries devastating consequences, including ~1.2 million deaths in 2019 alone. With significant public health resources required for the COVID-19 pandemic, the global TB burden will likely only increase in the coming years^1,2^. Meningitis is the most severe complication of TB, carrying a 50–60% mortality rate in persons living with HIV^3^. The diagnosis of TB meningitis (TBM) is notoriously difficult yet essential, as early and appropriate treatment is critical to prevent significant morbidity and mortality^4^. Due to the paucibacillary nature of TB infection in the central nervous system (CNS), culture and nucleic acid detection (e.g., TB PCR) are insensitive diagnostic tools^4–7^ compared to the TBM Uniform Case Definition (UCD), which was created to standardize research studies^8^. However, the clinical, radiological, and laboratory criteria that comprise the UCD lack specificity^9^. Thus, there is a double-edged problem of patients with delayed or missed diagnoses of TBM due to the inadequate sensitivity of available diagnostic tests as well as patients inappropriately diagnosed and empirically treated for TBM based on nonspecific clinical, laboratory, and radiologic criteria who have other infectious (and non-infectious) causes of meningitis^10–12^.

Metagenomic next-generation sequencing (mNGS) is a validated diagnostic assay for neuroinfectious diseases that can test for a wide variety of infections by amplifying all the genetic material (host and pathogen) from a cerebrospinal fluid (CSF) sample^13^. We and others have published case reports and case series demonstrating that CSF mNGS can diagnose TBM (and other infections that can clinically mimic TBM). These studies suggest that mNGS is specific but only moderately sensitive for detecting the small amounts of TB nucleic acid in the CSF of patients with TBM^10,11,14–16^. Here, we investigated the clinical utility of CSF mNGS in a large population of prospectively enrolled Ugandan adults living with HIV and suspected TBM. We sought to enhance test performance by leveraging the human gene expression component of the CSF mNGS data to develop a complementary machine learning classifier (MLC) that categorizes patients as having TBM or not. While host-based MLCs derived from blood and respiratory fluids are increasingly available to distinguish between infectious and non-infectious diseases (including TB)^17–21^, the picogram quantities of RNA in a typical CSF sample have thus far stymied the development of analogous host-based classifiers for patients with neuroinflammatory disease.

## Results

### Cohort demographics

From 2018–2019, 368 patients consented for the study. The median age was 35 years [IQR 29–41 years] with 42.7% female. Among the patients for whom the CD4 cell count was measured (*n* = 95), the median was 41 cells/mm^3^ [IQR 13–80 cells/mm^3^]. At the time of admission, 181 patients (49.1%) were documented as being on antiretroviral therapy (ART), with 41/181 (22.6%) of these patients known to have commenced ART in the prior month. Median duration of headache prior to admission was 14 days [IQR 7–21 days]. Median Glasgow Coma Scale (GCS) at presentation was 14 [IQR 14–15], 185/362 (51.1% of those with known GCS) had GCS < 15. Mortality at discharge or last contact was 25.9% (94/362) (Table 1).

**Table 1:** Baseline demographics by final diagnosis.

	Overall		Microbiological TBM Dx (TBM definite)		Uniform case definition TBM Dx (TBM probable)		Microbiologically positive non-TB meningitis (CM, ABM, VM)		Possible TBM, Indeterminate^a^	
No. participants	368		39		24		201		104	

Demographics	*N*	Median [IQR] or *N* (%)	*N*	Median [IQR] or *N* (%)	*N*	Median [IQR] or *N* (%)	*N*	Median [IQR] or *N* (%)	*N*	Median [IQR] or *N* (%)
Age (years)	368	35 [29, 41]	39	34 [29, 37]	24	35 [27, 39]	201	35 [29, 42]	104	35 [28, 42]
Female	368	157 (42.7%)	39	19 (48.7%)	24	10 (41.7%)	201	78 (38.8%)	104	50 (48.1%)
Headache duration, days	312	14 [7, 21]	30	14 [14]	19	14 [7, 30]	182	14 [7, 21]	81	14 [7, 30]

Baseline blood results	*N*	Median [IQR] or *N* (%)	*N*	Median [IQR] or *N* (%)	*N*	Median [IQR] or *N* (%)	*N*	Median [IQR] or *N* (%)	*N*	Median [IQR] or *N* (%)
CD4 + cell count	95	41 [13, 80]	16	76 [46, 117]	9	41 [30, 270]	67	27 [11, 66]	3	260 [45, 340]

Baseline CSF results	*N*	Median [IQR] or *N* (%)	*N*	Median [IQR] or *N* (%)	*N*	Median [IQR] or *N* (%)	*N*	Median [IQR] or *N* (%)	*N*	Median [IQR] or *N* (%)
CSF white blood cells per μL	343	4 [4, 65]	37	80 [4, 240]	23	4 [4, 250]	185	4 [4, 60]	98	4 [4, 4]
CSF WBC count >5	343	117 (34.1%)	37	25 (67.6%)	23	11 (47.8%)	185	61 (33.0%)	98	20 (20.4%)
CSF Lymphocyte, %	107	100 [90, 100]	23	100 [83, 100]	10	100 [86, 100]	57	100 [90, 100]	17	100 [100, 100]
CSF total protein, mg/dL	322	77 [30, 131]	35	184 [99, 211]	18	107 [73, 164]	178	75 [38, 112]	91	40 [23, 104]
CSF glucose, mg/dL	247	54 [30, 78]	31	23 [18,44]	17	44 [25, 83]	132	53 [32, 76]	67	68 [45, 104]

Clinical	*N*	Median [IQR] or *N* (%)	*N*	Median [IQR] or *N* (%)	*N*	Median [IQR] or *N* (%)	*N*	Median [IQR] or *N* (%)	*N*	Median [IQR] or *N* (%)
Prior tuberculosis diagnosis	367	72 (19.6%)	39	5 (12.8%)	24	2 (8.3%)	200	36 (18.0%)	104	29 (27.9%)
Alive at discharge or last contact	362	268 (74.0%)	38	26 (68.4%)	24	19 (79.2%)	196	139 (70.9%)	104	84 (80.8%)

Definite, probable, and possible TBM defined by the TBM Uniform Case Definition (Supplementary Table 1). Not-TBM group comprised of patients with confirmed infections other than TB.

*TBM* tuberculous meningitis, *CM* cryptococcal meningitis, *ABM* acute bacterial meningitis, *VM* viral meningitis, *IQR* interquartile range, *CSF* cerebrospinal fluid.

^a^*n* = 9 ‘Other’ participants were missing Uniform Case Definition for TBM. Six had cryptococcal antigenemia.

CSF was collected for mNGS from 368 patients. Of note, a second CSF sample was available for 14 patients, and results are represented as pathogens detected per patient, not per sample. Basic CSF studies (i.e., cell counts, chemistry) are listed in Table 1. One hundred eighty (48.9%) were diagnosed with cryptococcal meningitis (CM) based on: a positive serum cryptococcal antigen (CrAg) as well as a positive CSF CrAg and/or positive fungal culture (Not-TBM group). 2.9% (11/368) of patients were diagnosed with bacterial meningitis (Not-TBM group). 10/366 (2.7%) were diagnosed as viral meningitis (Not-TBM group).

Sixty-three patients (17.1%) were classified as probable or definite TBM, of which 61.9% (39/63) had definite TBM and 38% (24/63) were classified as probable TBM. Definite TBM was diagnosed by Xpert MTB/RIF Ultra in 97.4% (38/39) of cases. 66.6% (12/18) of cases were TB culture positive (11/18 cases were positive for TB by both Xpert MTB/RIF Ultra and culture). 16.3% (60/368) of cases were classified as possible TBM. 11.9% (44/368) were categorized as indeterminate. Nine cases had missing data for their TBM UCD score, 6 of these cases had cryptococcal antigenemia, and 3 cases had a final hospital diagnosis listed as unknown. These patients were classified as indeterminate.

### Training and test cohorts

Two hundred forty patients were included in the training cohort, ~2/3 of the cohort. Within this cohort, 70 samples had either definite TBM or some other neurological disease (OND) (see Methods) and were used for the training of the MLC. The learning curves generated show that further addition of samples would have diminishing improvements to performance of the classifier (Supplementary Fig. 1). One hundred thirty patients were included in the test cohort. Apart from two cases, cases were exclusive to either the training or test cohorts. In one case, the CSF was used in both the training and test cohorts, and in the other case, CSF collections from 2 separate days from a single case were split between the training and test cohorts. In the training cohort, the percentage of cases with definite TBM was 12.6% (*n* = 30) whereas in the test cohort, it was 6.3% (*n* = 8) (Supplementary Table 1).

### Training cohort mNGS results

Median sequencing depth for the RNA-Seq libraries after removal of External RNA Control Consortium (ERCC) sequences (both through bioinformatic filtering and depletion of abundant sequences by hybridization (DASH))^22,23^ was 5,668,087 paired-end reads (IQR 2,804,906–13,858,372). The median RNA input mass was 4.37 pg (0.8–1326.4 pg). The median sequencing depth for the DNA-Seq libraries was 27,341,015 (IQR 19,161,771–32,158,040). Transcripts aligning to an average of 3212 protein-coding genes (113–5335) were detected. We did not observe a strong correlation between the total number of protein-coding genes for which transcripts were detected in each sample and RNA mass, cell count, and library preparation method (i.e., with or without ERCC DASH) (Supplementary Fig. 2A).

### TBM detection by mNGS

We evaluated all definite TBM cases that were mNGS positive for TB. The median number of sequences aligning to the TB genome was 24 in the DNA-seq libraries (2–552 sequences) (median 1.4 reads per million reads (rpM) sequenced, 0.1–25.2 rPM), and 3683 in the RNA-seq libraries (1–116,083 sequences) (median 155.7 rPM, 0.1–9050 rpM). No TB reads were detected in the 8 “no template” water controls. Given the very low abundance of TB reads in definite TBM CSF and the lack of any TB sequences detected in the “no template” water controls, detection of TB by mNGS was defined as 1 or more sequences, with the entire sequence aligning (with no alignment to any other mycobacterial species) to the TB genome with ≥98% nucleotide sequence identity.

### Training cohort machine learning classifier

Seventy microbiologically proven samples (TBM: *n* = 32 and OND: *n* = 38) infections, identified in the training cohort, were used for the training of the MLC. Samples were sequenced across two batches (TBM: batch 1 (*n* = 12), batch 2 (*n* = 20); OND: batch 1 (*n* = 19), batch 2 (*n* = 19)) to mitigate batch effects. Sixteen thousand six hundred seventeen different iterations of the classifier were run to determine the optimal parameters: all combinations of five ML methods (with parameters) with/without feature selection, addition of the co-variate matrix variables before/after feature selection, class weights (TBM, OND) and prior count for logCPM. We wanted to identify a small set of features to distinguish the TBM versus OND, and hence chose L1 regularization. The first round of iterations (*n* = 16,050) of the classifier was bootstrapped 100 times. A second round of iterations (*n* = 567) to fine tune the classifier was run 1000 times. A cutoff of ≥50% was used for the diagnosis of TBM.

Two support vector machine (SVM) classifiers, with L1 regularization (regularization parameter C = 0.1), independent of the co-variate matrix, with highest sensitivities were chosen: MLC1 and MLC2. MLC1 had a prior count = 75, and top 50 features, and MLC2 had a prior count = 75, top 40 features and class weight for TBM = 2. This class weight of 2 further increased the sensitivity of TBM detection in our training cohort.

MLC1 had an area under the receiver operator characteristics curve (AUC) = 0.94, sensitivity = 0.84 and specificity = 0.95 on the training cohort. Of the 10 resequenced samples, MLC1 classified 4/5 TBM and 5/5 OND correctly. A total of 15 genes were used to classify TBM and 111 genes to classify OND (bootstrap = 1000) (Supplementary Data 1). For the TBM group, four genes were used predominantly (>500/1000 bootstrap) for classification: *FTL* (528/1000), *NFKBIA* (674/1000), *SOD2* (999/1000), and *GBP5* (1000/1000). For the OND group, three genes were used predominantly for classification: *EEF1A1* (532/1000), *TMSB4X* (544/1000), and *ACTB* (717/1000). We created two additional classifiers (SVM, C = 0.1, prior count = 75) based on these top genes to classify TBM vs. OND: a four-gene (*FTL*, *NFKBIA*, *SOD2*, and *GBP5*) and seven-gene (*FTL*, *NFKBIA*, *SOD2*, *GBP5*, *EEF1A1*, *TMSB4X*, and *ACTB*) classifier. In the main manuscript we only discuss results from MLC1. Performance of the MLC2, four-gene and seven-gene classifiers are described in the Supplementary Information (Supplementary Note 1).

### Test cohort mNGS and MLC detection of TBM

Median sequencing depth for RNA-seq after removal of ERCCs (both through bioinformatic filtering and DASH) was 2,576,270 (IQR 1,274,462–13,761,173). The median sequencing depth for DNA-seq was 21,698,038 (IQR 9,842,494–29,087,373) The median RNA mass was 7.03 pg (2.7–4750 pg).

There were nine cases of definite TBM and 13 probable TBM cases in the test cohort. mNGS detected TB in six of the definite TBM cases, for a sensitivity of 6/9 (66.6%). In the probable TBM group, mNGS detected one case of TB, one case of *Toxoplasma gondii*, one case of *C. neoformans*, one case of *T. gondii* and varicella zoster virus (VZV) co-infection, and one case of *T. gondii* and *C. neoformans* co-infection. The case with *T. gondii* and *C. neoformans* co-infection was a repeat CSF sample present in the training cohort which the authors were blinded to.

**Fig. 1:**
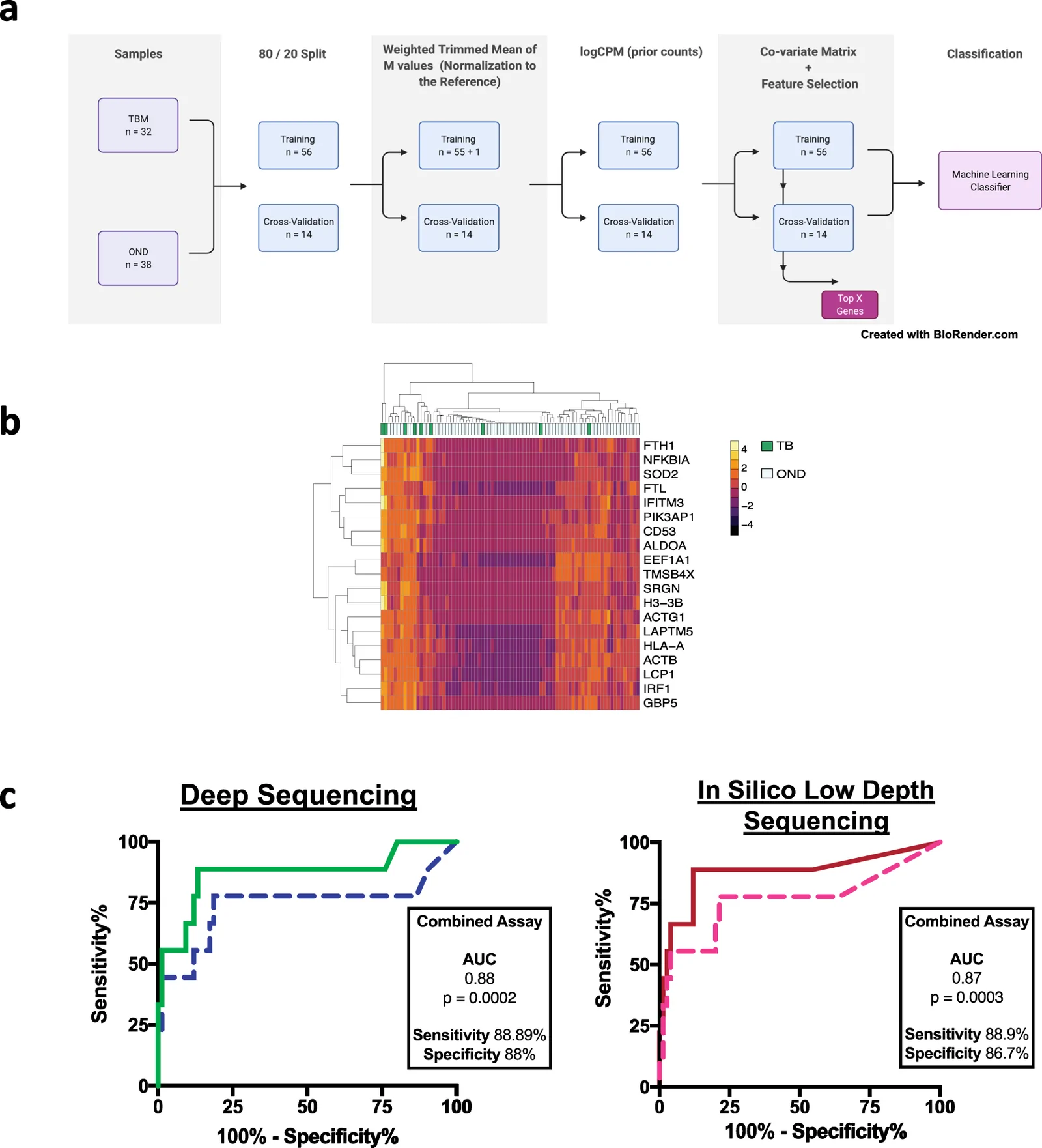
Development of a host-based machine learning classifier from cerebrospinal fluid RNA-seq data. **A** Workflow for creation of the machine learning classifier using 70 microbiologically proven samples, through PCR or conventional testing, identified in the training cohort. **B** Most predictive 4 genes of a 15 gene classifier to classify TBM vs OND. **C** Left: ROC curve for the combined mNGS and MLC assay. Blue dotted line is the MLC assay alone, and the green solid line is the combined assay. If the MLC categorized a case as TBM, but mNGS detected a non-TB pathogen, then mNGS overruled the MLC result, thus increasing specificity of the overall assay. Right: In silico prediction of shallow depth (i.e., 100,000 reads for the RNA-seq library and 500,000 reads for the DNA-seq library) sequencing results. Pink dotted line is the MLC assay alone, red solid line is combined assay with mNGS. Current cost prediction for shallow depth sequencing in $75 per patient. CPM counts per million, GBP5 guanylate binding protein 5, FTL ferritin light chain, NFKBIA NF-kappa-B inhibitor alpha, SOD2 superoxide dismutase 2, TBM tuberculous meningitis, OND other neurological disease, MLC machine learning classifier, mNGS metagenomic next-generation sequencing, AUC area under the receiver operator curve. Source data are provided as a Source Data file.

The MLC correctly called seven out of nine cases of definite TBM as TBM for a sensitivity of 77.7%. The MLC called three cases as TBM in the probable TBM group. One of these cases had TB detected by mNGS (Fig. 1), and the other two cases had no pathogen identified. We compared the MLC failures and successes and saw no statistically significant differences regarding their RNA mass, CSF cell counts or the number of protein-coding genes with non-zero counts (Supplementary Fig. 2B).

### mNGS results for entire cohort

Sequences aligning to *M. tuberculosis* were detected by RNA-seq, DNA-seq or both in 37 cases throughout the cohort (Supplementary Table 2). TB was detected by mNGS in 74.4% (29/39) of definite TBM cases. TB was detected by mNGS in 12.5% (3/24) of cases classified as probable TBM (i.e., negative CSF TB PCR and TB culture). Thus, overall sensitivity for detecting TB by mNGS in cases defined as definite or probable TBM was 50.8% (32/63). Sensitivity increased to 54.2% (32/59) when excluding cases from the probable TBM group in which mNGS detected alternate infections. TB was detected in 3.8% (4/104) of cases classified as possible TBM or indeterminate. TB was identified as a co-infection in one patient with CM (Not-TBM group) for whom TB culture and Xpert Ultra had not been performed. Regarding alternate infections, one definite TBM case had a *T. gondii* co-infection detected by mNGS. As stated, mNGS detected four cases with non-TB infections in the probable TBM group, including three cases previously mentioned in the test cohort results section (*C. neoformans* in one case, *T. gondii* in one case and a *T. gondii* and VZV co-infection in another), and one case of a *T. gondii* and *C. neoformans* co-infection in the training cohort.

### Viral infections

**Fig. 2:**
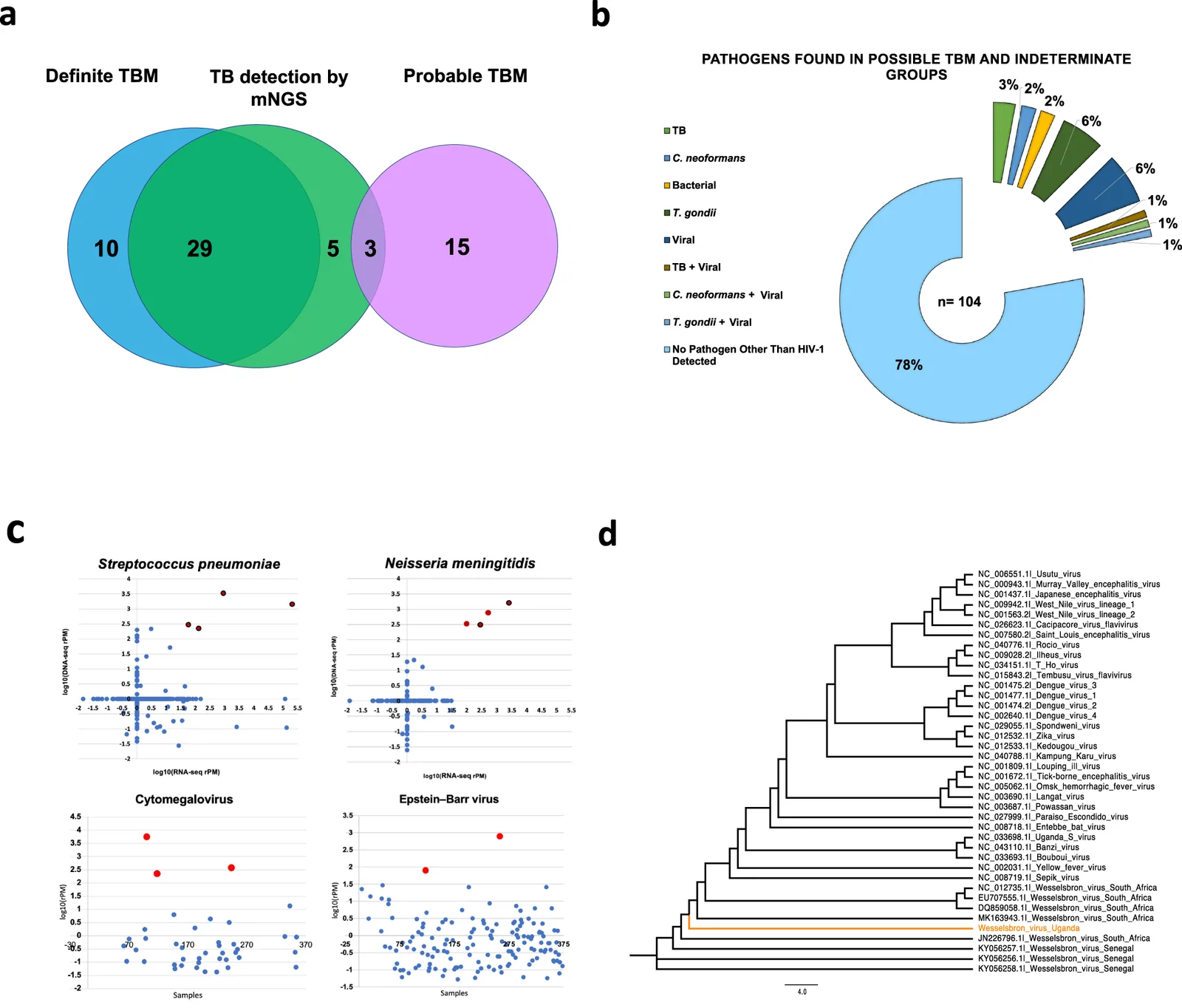
Pathogen detection by mNGS. **A** Detection of TB for entire cohort. Blue circle represents definite TBM (i.e., detected by GeneXpert Ultra and/or culture). Pink circle represents probable TBM cases based on clinical consensus but GeneXpert Ultra and/or culture negative. Green circle is TBM detected by mNGS. Five additional cases of TBM were detected in possible TB, indeterminate and Not-TBM groups. **B** Breakdown of pathogens found in possible TBM and indeterminate groups. **C** Top two graphs are log10(rPM) for normalized DNA-seq and RNA-seq reads to *Streptococcus pneumoniae* and *Neisseria meningitidis* detected in every sample sequenced. Red dots indicate samples with greater than 1 log fold abundance greater than the mean cohort abundance in both the DNA-seq and RNA-seq datasets relative to the remaining cohort. Red dots circled in black indicate samples that were diagnosed as bacterial meningitis during hospital admission in Uganda. Bottom two graphs demonstrate a similar method performed for Epstein-Barr virus and cytomegalovirus as both these viruses can be seen in low abundance in neuroinflammatory conditions. Red dots indicate samples with Epstein-Barr virus or cytomegalovirus with rPM 2 log fold higher than the mean cohort abundance in DNA-seq. **D** Phylogenetic tree for Wesselsbron virus detected in 1 patient in the possible TBM group. Phylogenetic tree was built with a multiple sequence alignment using the assembled genome and reference genomes obtained from National Center for Biotechnology Information. The best-fitting evolutionary model was picked by ModelTest-NGv0.1.5, and a phylogenetic tree was built using RAxML-ng v0.6.0. Scale bar indicates length associated with 4.0 nucleotide substitutions. TBM tuberculous meningitis, TB *Mycobacterium tuberculosis,* mNGS metagenomic next-generation sequencing, RNA-seq ribonucleic acid sequencing, DNA-seq deoxyribonucleic acid sequencing, rPM reads per million. Source data are provided as a Source Data file.

Nineteen neuroinvasive viruses (other than HIV-1) were detected by mNGS across the entire cohort (Table 2). As described above, VZV was detected as a co-infection with *T. gondii* in a case classified as probable TBM. Ten viruses were detected in the possible TBM and indeterminate groups (herpes simplex virus type 1 (HSV-1) *n* = 1, HSV-2 *n* = 1, VZV *n* = 3, cytomegalovirus (CMV) *n* = 2, human parvovirus B19 *n* = 1, rubella virus *n* = 1, Wesselsbron virus *n* = 1). Wesselsbron virus, a neuroinvasive flavivirus endemic to sub-Saharan Africa^24,25^, was identified along with TB (see Supplementary Information for clinical history). mNGS yielded 5739.5 rPM with 96.1% coverage across the Wesselsbron virus genome (Fig. 2D and Supplementary methods for phylogenetic tree).

**Table 2:** All non-TBM pathogens detected in entire cohort.

Patient	Pathogen	rPM (DNA/RNA)	Orthogonal confirmation	Group	Co-infection	Cohort
1	HSV-1	391.4	PCR	Possible TBM/Indeterminate		Training
2	HSV-2	189	PCR^a^	not-TBM		Training
3	HSV-2	8.1	PCR	Possible TBM/Indeterminate	*T. gondii*	Training
4	VZV	4660.1	PCR	Possible TBM/Indeterminate		Training
5	VZV	6823.1	PCR	Possible TBM/Indeterminate		Training
6	VZV	7065.4	PCR	Probable TBM	*T. gondii*	Test
7	VZV	21.8	PCR	not-TBM	CM	Training
8	VZV	5.1	PCR^a^	not-TBM		Training
9	VZV	741.9	PCR	Possible TBM/Indeterminate		Test
10	CMV	5607.5	PCR	Possible TBM/Indeterminate		Training
11	CMV	223.2	PCR^a^	not-TBM	CM	Training
12	CMV	377.9	PCR	Possible TBM/Indeterminate		Test
13	Parvovirus B19	181.6	PCR	Possible TBM/Indeterminate	CM	Training
14	Parvovirus B19	78.5	PCR	not-TBM		Training
15	Measles virus	6663.2	mNGS	not-TBM	CM	Training
16	Wesselsbron virus	5739.5	mNGS	Possible TBM/Indeterminate	TB	Training
17	Rubella virus	37.3	mNGS	Possible TBM/Indeterminate		Training
18	EBV	787.3	mNGS	not-TBM		Test
19	EBV	80	mNGS	not-TBM		Test
20	JCV	1.5	mNGS	not-TBM	CM	Training
21	*Nocardia brasiliensis*	465.5	mNGS	Possible TBM/Indeterminate		Training
3	*T. gondii*	3799.7	PCR	Possible TBM/Indeterminate	HSV-2	Training
22	*T. gondii*	2305.4	PCR	Definite TB	TB	Training
23	*T. gondii*	1838.9	PCR	Possible TBM/Indeterminate		Test
24	*T. gondii*	242.4	PCR	Probable TBM	CM	Training
25	*T. gondii*	653.7	PCR	not-TBM	CM	Training
26	*T. gondii*	847.1	PCR	Possible TBM/Indeterminate		Training
27	*T. gondii*	362.3	PCR	Possible TBM/Indeterminate		Training
28	*T. gondii*	355.4	PCR	not-TBM	CM	Training
29	*T. gondii*	572.5	PCR	Possible TBM/Indeterminate		Test
30	*T. gondii*	626.7	PCR	Possible TBM/Indeterminate		Test
31	*T. gondii*	369.1	PCR	not-TBM	CM	Test
6	*T. gondii*	251.3	PCR	Probable TBM	VZV	Test
32	*T. gondii*	31.9	PCR	not-TBM	CM	Training
33	*T. gondii*	4960	PCR	Probable TBM		Test
34	*T. gondii*	4956.2	PCR	Possible Indete/Indeterminate		Test
35	*S. pneumoniae*	232.1/644.4	mNGS	not-TBM		Training
36	*S. pneumoniae*	4015.8/4381.1	PCR^a^	not-TBM		Training
37	*S. pneumoniae*	1728.4/868211.2	PCR^a^	not-TBM		Training
160	*S. pneumoniae*	66.0/296.1	mNGS	Not_TBM		Training
38	*N. meningitidis*	365.2/930.3	mNGS	not-TBM		Test
39	*N. meningitidis*	1964.1/22646.3	PCR^a^	not-TBM		Test
40	*N. meningitidis*	894.3/1144.5	mNGS	Possible Indete/Indeterminate		Test
41	*N. meningitidis*	402.1/767.6	mNGS	not-TBM	CM	Test

*HSV* herpes simplex virus, *VZV* varicella zoster virus, *CMV* cytomegalovirus, *EBV* Epstein-Barr virus, *JCV* JC virus, *PCR* polymerase chain reaction, *mNGS* metagenomic next-generation sequencing, *TB* *Mycobacterium tuberculosis*, *CM* cryptococcal meningitis.

^a^Orthogonal testing confirmed with Biofire.

Nine additional viruses were detected in the Not-TBM group: HSV-2 *n* = 1, VZV *n* = 2, CMV *n* = 1, human parvovirus B19 *n* = 1, measles virus *n* = 1, John Cunningham (JC) virus *n* = 1, and Epstein-Barr virus (EBV) *n* = 2 were detected as co-infections in patients with CM (Fig. 2C). In addition, viral reads to likely nonpathogenic viruses were detected (Supplementary Data 2).

### Bacterial infections

We identified nine cases of bacterial meningitis, including four cases of *Streptococcus pneumoniae* (Fig. 2C). All four of the cases were categorized as acute bacterial meningitis by hospital discharge diagnosis (Not-TBM group) (Table 2). One case of *Nocardia brasiliensis* was detected by CSF mNGS in a patient classified as possible TBM. Four cases of *Neisseria meningitidis* were detected. Two of these cases had a clinical diagnosis of bacterial meningitis (Not-TBM group). One case was in the possible / indeterminate group, and the other case was a co-infection in a patient with CM (Not-TBM group). mNGS also detected *C. neoformans* in this case (9.21 rPM).

### Parasitic infections

Fifteen cases of *T. gondii* were identified. One case was identified as a co-infection in a patient with definite TBM, three cases were identified in patients classified as probable TBM, one case which also had *C. neoformans* identified on mNGS (CSF CrAg negative), and another with a co-infection with VZV (as described above). Six cases of *T. gondii* were identified in patients classified as possible TBM, and four were identified as co-infections in patients with CM (Table 2).

### A combined microbial and host-based MLC assay for diagnosing TBM and its mimics in a Ugandan HIV-positive cohort

The host MLC alone classified cases in the blinded test set as TBM vs OND with a sensitivity of 77.8% (CI: 40–97.2%) (7/9) sensitive and 76% (CI: 64.8–85.1%) (57/75) specific with an AUC of 0.74 (*p* = 0.02). The combined mNGS and host MLC displayed 88.9% (CI:51.8–99.7%) (8/9) sensitivity, 86.7% (CI:76.8–93.4%) (65/75) specificity, with an AUC of 0.87 (*p* = 0.0003). Concordance was 50% (11/22) against the UCD of definite and probable TBM and 61% (11/18) when cases of non-TB pathogens detected in the probable group were excluded. The combined assay detected three cases of TB in the probable TBM group and eight additional TBM cases initially classified as possible TBM. Improvement in specificity of the combined assay from the standalone host MLC classifier was due to eight cases that were classified as TBM by the MLC but were found to have other pathogens detected on mNGS, which overruled the MLC classification. These were three cases of *N. meningitidis*, three cases of *C. neoformans*, one case of VZV, and one case of EBV. One case of definite TBM had lumbar punctures (LPs) performed on days 1 and 3 post-initiation of TBM treatment. Day 1 CSF was used as part of the MLC training cohort, and TB was detected by mNGS. Day 3 CSF was used in the test cohort to which the authors were blinded. mNGS failed to detect TB in the day 3 CSF; however, the MLC still classified this sample as TBM. We considered this a failure of TB detection by mNGS when analyzing the test cohort data.

### In silico evaluation of mNGS and host gene expression MLC with shallow depth sequencing for low resource settings

Shallow depth sequencing of the test set had no impact on MLC sensitivity or specificity: MLC1 correctly identified 7/9 definite TBM cases (100% concordance with deep sequencing MLC results) as TBM, and 59/75 OND cases as OND at all subsampled depths down to 100,000 reads. The final AUC, sensitivity, and specificity for the MLC1 were calculated once the optimal threshold was determined for the mNGS assay.

### mNGS subsampling

mNGS results from the original samples were used as a baseline for comparison at various subsampling depths. DNA-seq libraries in which TB was detected for the entire cohort at the original sequencing depth (*n* = 25) had TB detected with 56% sensitivity (14/25) at 2,000,000 reads and 32% sensitivity at 500,000 reads (8/25). RNA-seq libraries in which TB was detected for the entire cohort at the original sequencing depth (*n* = 9) had TB detected with 100% sensitivity (9/9) at a subsampling depth of 2,000,000 reads and 88.9% sensitivity (8/9) at 100,000 reads (Supplementary Table 3).

For non-TB pathogens, 15 DNA and 6 RNA samples consisting of viral, bacterial, and parasitic infections were analyzed, including one sample with a co-infection. For DNA-seq, at a subsample depth of 500,000 reads, mNGS detected 100% of the pathogens that were detected at the original sequencing depth. At the lowest subsample depth of 100,000 reads, 13/15 (86.67%) of pathogens were detected. When subsampling RNA-seq libraries at a depth of 2,000,000 or 100,000 reads, 6/6 (100%) of pathogens detected at the original sequencing depths were detected (Supplementary Table 3).

Based on these results, the optimal sequencing depth for the detection of non-TBM pathogens was considered 100,000 paired-end reads for RNA-seq and 500,000 pair end reads for DNA-seq. While TB detection by mNGS at shallow sequencing depths was insensitive, the MLC classifier still maintained similar sensitivity to classify TBM at 100,000 paired-end reads. We therefore reran all microbiologically proven samples from the final test cohort (*n* = 84) at 100,000 reads for RNA-seq and 500,000 reads for DNA-seq for through the MLC and the open source, cloud-based metagenomics pipeline (Chan Zuckerberg ID or CZID)^26^ pipeline to assess AUC, sensitivity and specificity of our in silico low depth sequencing model. The MLC had an AUC of 0.76 (*p* = 0.01) with a sensitivity of 77.8 (CI:40–97.2%) and a specificity of 78.7 (CI:67.8–87.3%). The combined MLC and mNGS assay had an AUC of 0.88 (*p* = 0.0002) with a sensitivity of 88.9% (CI:51.8–99.7%) and a specificity of 88% (CI:78.4–94.4%) (Fig. 1C).

### Cost analysis

At a depth of 100,000 reads for the RNA library and 500,000 reads for the DNA library, one sample would require 600,000 total paired-end reads. An Illumina iSeq generates 8,000,000 paired-end reads per flow cell. Thus, at 600,000 reads per sample, it would be possible to evaluate 13 samples/run. The current cost of the iSeq cartridge at the time of writing was $USD495, or $38 per sample. With our current library preparation protocol, the total cost of reagents would be $28.25/sample at a 0.5× reaction volume with the NEBNext® Ultra™ II kits: NEBNext Ultra II RNA library kit (NEB Cat No. E7770) $15.75/sample, NEBNext® Ultra™ II FS DNA Library Prep Kit (E7805L) $12.50/sample. SPRIselect 450 mL reagent kit (B23319) $5/sample, and QIAseq FastSelect—rRNA HMR Kit (334386) at 1:100 dilution $4.60/sample. The overall cost/sample would be $75.85, excluding the upfront costs of the Illumina iSeq and ongoing service contracts.

## Discussion

Characterizing the epidemiology of infectious and non-infectious etiologies of meningitis and encephalitis is inherently challenging and only compounded by the limited diagnostic capacity in sub-Saharan Africa^27–29^. Because of the high prevalence of advanced HIV-1 and TB infections in Uganda*, C. neoformans* and TB are the most commonly diagnosed causes of meningitis. Yet, the etiology of many other cases of meningitis and/or encephalitis is never known^30^. Here, we utilized unbiased CSF mNGS to obtain a more comprehensive profile of neurologic infections (and co-infections) in Ugandan adults with subacute meningitis living with HIV. We identified many treatable and vaccine-preventable infections. In addition, we describe the first use of human transcriptomic data generated by a CSF mNGS assay to combine direct detection of pathogens with a host-based MLC that identifies patients with or without TBM.

The diagnosis of TBM remains difficult due to the paucibacillary nature of the disease. While mNGS can detect TB in CSF, mNGS still has limited sensitivity, akin to TB PCR^14–16^. Here, mNGS demonstrated 74.4% concordance against microbiologically confirmed cases of TBM (i.e., definite TBM) and 54.2% concordance against the UCD of definite and probable TBM after removing probable TBM cases in whom mNGS identified an alternate infection. We detected an additional five cases of TBM missed by conventional testing in the possible TBM and indeterminate groups and a sixth case in a patient diagnosed with CM (Not-TBM group), who did not undergo conventional testing for TBM.

mNGS identified 43 additional neurological infections that were either sole or co-infections, including neuroinvasive viruses like Wesselsbron virus, a flavivirus endemic to the region that mainly infects livestock^24^ but with several reports of human infections, including neuroinvasive disease^25,31^. This virus was present in a patient co-infected with TB (Supplementary Information). We also identified 14 cases of treatable viruses (HSV-1, HSV-2, VZV, CMV, and EBV) and 2 vaccine-preventable viruses (measles virus and rubella virus). Measles can present with varying CNS manifestations, including measles inclusion body encephalitis occurring in HIV-infected patients^32^. Two cases of human parvovirus B19 were detected. Neurological manifestations of human parvovirus B19 have been previously described in both immunocompetent and immunocompromised patients^33^. In addition to viral infections, we found 15 cases of CNS toxoplasmosis and 1 case of CNS *Nocardia*, highly morbid but treatable conditions if diagnosed early^34,35^.

Despite the relatively pauci-cellular content of many CSF samples in this cohort of immunocompromised patients, we recovered a rich human gene expression dataset, detecting transcripts to an average of more than 3000 genes/sample. Leveraging these data, we built a sensitive MLC to distinguish TBM from ONDs that can mimic TBM. Five of six definite TBM cases in the test cohort were classified correctly, improving upon the direct detection of TB in 3/6 cases by mNGS. Our MLC primarily uses four genes to classify TBM: *GBP5*, *SOD2*, *NFKBIA*, and *FTL*. *GBP5*, which promotes NLRP3 inflammasome responses to pathogenic bacteria^36,37^, and is part of a 3 gene signature (*GBP5*, *DUSP3*, and *KLF2*) that distinguishes active pulmonary TB from other infections^38,39^. Additional studies using GBP5 have also distinguished TB from non-TB pneumonia with >90% sensitivity and specificity^40,41^. The three other primary genes (*SOD2*, *NFKBIA*, and *FTL*) used in our MLC have not been described in other TB host gene expression signatures. Interestingly, SOD2-mediated acidification of phagosomes has been shown to promote survival of TB in their host and serves as a marker for oxidative stress^42–44^. Ferritin has been recognized as an important factor in host immunity against TB, and ferritin heavy chain in particular has been shown to protect against TB in murine studies^45^. *NFKBIA* is a member of the NF-kappa-B (NF-κB) inhibitor family. NF-κB signaling dynamics, triggered via tumor necrosis factor-α binding to receptors on macrophages, has been shown to play a key role for survival of TB^46^. Particularly, Bai et al., reported that NF-κB inhibition, decreased survival of TB in macrophages^47^.

Overall, the MLC was optimized for high sensitivity, but there was a trade-off with specificity. The MLC performed poorly classifying OND cases that had infections which were not present in the training cohort. For instance, three of four cases of *N. meningitidis* were classified as TBM. Fortunately, coupling case classification by the host MLC with direct detection of pathogens by mNGS enhanced the overall specificity of the assay. In other words, direct detection of a pathogen by mNGS overruled the MLC if a pathogen other than TB was detected. Training the MLC on a larger cohort of patients with a wider range of ONDs will further improve accuracy.

Implementing these technologies in low resource settings is critical to improve prospective diagnoses and the institution of appropriate therapies. The utility of our diagnostic tool is not only its ability to detect TB, but also co-infections and infectious TB mimics with enhanced sensitivity for TB enabled by the host MLC analysis whose data are generated as part of the mNGS assay. Our in silico analyses suggest that only an average of 600,000 paired-end reads are required to achieve results that we and others have traditionally achieved with millions or tens of millions of reads/sample^10,14,48^. In addition to an estimated per sample cost of $75, the cost-effectiveness of this assay will likely be enhanced in low resource settings lacking numerous pathogen-specific PCR assays and other diagnostic modalities commonly at the disposal of clinicians in high resource settings. Thus, mNGS, may serve as a “leapfrog” technology in this context.

This study has several additional limitations. It remains to be determined how well the genes that comprise the MLC translate to immunocompetent individuals though we are heartened that the key genes in our classifier have significant overlap with host transcriptomic studies in pulmonary TB, including non-HIV-positive patient populations^39^. In addition, host responses may vary between patients with different genetic backgrounds and based on the virulence of the infecting TB strain. Although almost all LPs were performed prior to initiation of therapy, we did not have data about prehospital antibiotic treatment, which may have impacted CSF pathogen load and host gene expression profiles. As illustrated in the case vignettes (Supplementary Information), we made every attempt to clinically correlate candidate pathogens detected by mNGS (in addition to confirmatory laboratory testing). However, the lack of widely available advanced neuroimaging and other testing modalities limited clinical adjudication to some degree. This is reflected in the fact that we detected high levels of HIV in the CSF of many patients, some of whom likely had HIV-associated dementia. However, we were not able to confidently classify them as such. Additionally, while we had a large cohort of patients, the number of cases of definite TBM in the training and test cohorts was small which limited the precision of the point estimates for the sensitivity, specificity, and AUC of the combined assay.

Currently mNGS is an expensive technology; however, research is underway to make this tool more accessible, especially for low resource settings where the burden of infectious diseases is high and the availability of many pathogen-specific assays is low^48–50^. Here, we demonstrated that mNGS identified many previously undiagnosed but treatable neurologic infections, even in patients already diagnosed with one cause of infectious meningitis. In addition, we developed a first-of-its-kind combined diagnostic assay utilizing mNGS and analysis of the CSF host transcriptome to diagnose TBM vs other non-TB meningitis etiologies. The accuracy of this method will only increase with the incorporation of larger patient numbers and holds the promise of more sensitively and rapidly detecting TBM along with TBM co-infections and TBM mimics, many of which are treatable or vaccine-preventable illnesses. Lastly, the ability to generate useful host transcriptomic data from pauci-cellular CSF augurs well for developing future MLCs that distinguish between other clinically overlapping neuroinflammatory syndromes like viral and autoimmune encephalitis.

## Methods

### Study cohort

**Fig. 3:**
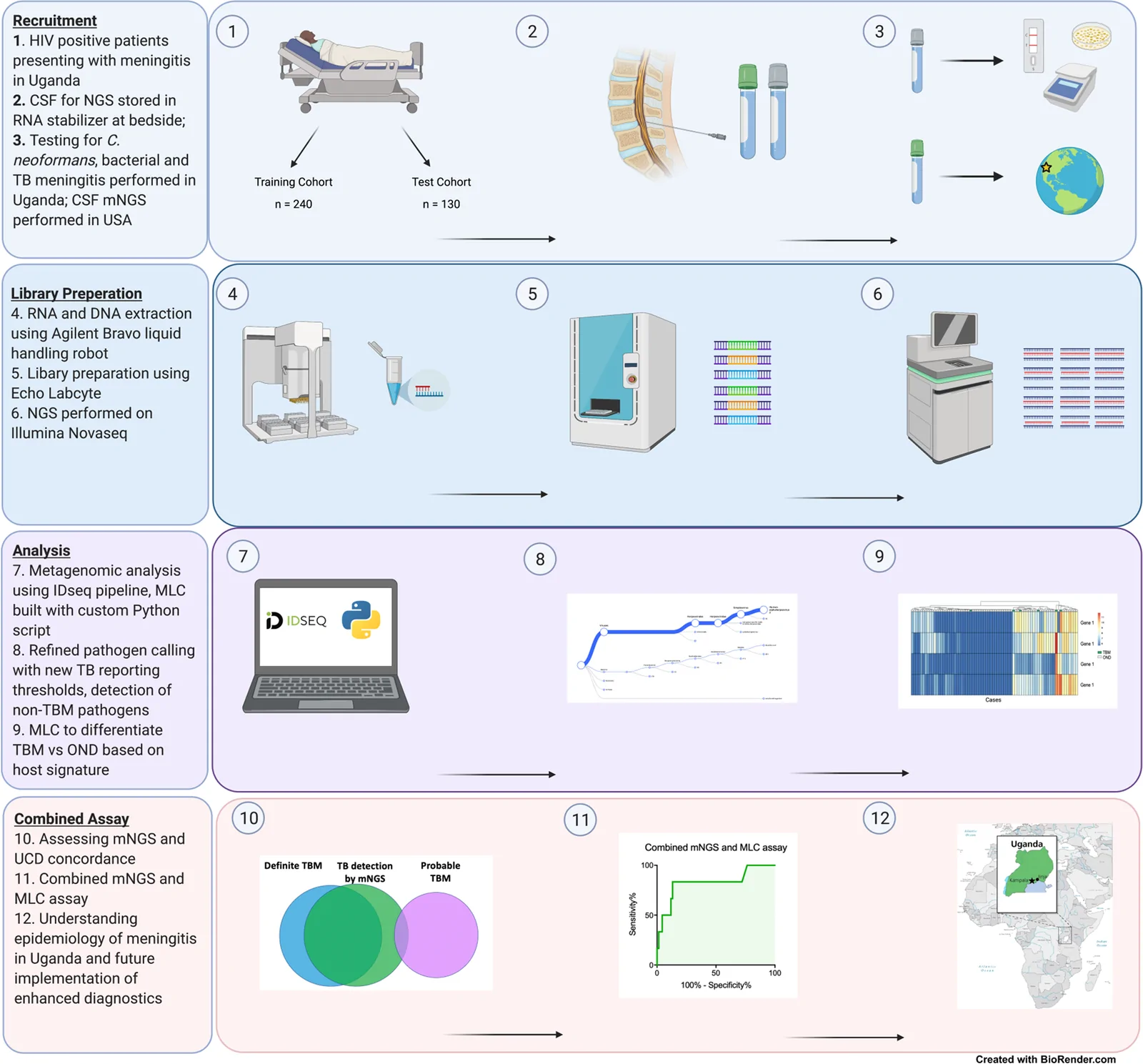
Study workflow. Four major components of the study: recruitment, library preparation, analysis, and creation of the combined assay. HIV human immunodeficiency virus, CSF cerebrospinal fluid, NGS next-generation sequencing, mNGS metagenomic next-generation sequencing, USA United States of America, RNA ribonucleic acid, DNA deoxyribonucleic acid, MLC machine learning classifier, TB *Mycobacterium tuberculosis,* OND other neurological disorders, UCD uniform case definition.

All research was conducted complies with all ethical regulations. Patients were prospectively recruited as part of the “Improving Diagnostics and Neurocognitive Outcomes in HIV/AIDS-related Meningitis” study (NCT01802385), a prospective cohort study underway in Uganda. Although some patients studied here later enrolled into other unrelated clinical trials, the analyses in this study of baseline CSF specimens are not related to the outcomes of any therapeutic intervention. Research was approved by University of Minnesota (IRB Study ID STUDY00006856), Infectious Diseases Institute at Makerere University and the Mulago Hospital Research and Ethics Committee (IRB Study ID MHREC1246), and University of California San Francisco (IRB 13–12236). All HIV-positive patients presenting with signs and symptoms concerning for meningitis (presentation with some combination of headache, fever, nuchal rigidity, neurologic deficit, or altered mental status) to Kiruddu Regional Referral Hospital, Kampala, Uganda from March 2018 to March 2020 were screened for study inclusion. LP was performed during days 1–3 of admission utilizing a standardized diagnostic algorithm. All patients had extensive demographic, clinical, biochemical, and microbial data collected. However, CD4 + T cell counts and HIV viral loads were not available for the majority of patients. Clinical data included presenting signs and symptoms, prior TB history and prophylaxis, response to antimicrobial and adjunctive treatments, and hospital discharge status. Baseline CSF cell count, protein, glucose, microscopy, gram stain, bacterial cultures, CrAg, and fungal culture were obtained for all participants. If the CrAg was positive, the patient was treated for CM, and no further microbiological diagnostic testing was performed. If the CSF CrAg was negative, CSF Gene Xpert MTB/RIF Ultra and TB culture were performed to evaluate for TBM^8^. If TB was detected by either of these tests, the patient was considered to have definite TBM. The BioFire^®^ FilmArray^®^ Meningitis/Encephalitis (ME) Panel was performed on a subset (*n* = 52) of CSF samples, regardless of presumed diagnosis based on the clinician’s discretion. For all enrollees, ~1 mL of CSF was collected in Zymo DNA/RNA Shield collection tubes (Zymo Research; Irving, CA) and subsequently frozen at −70 °C within 8 h of collection and shipped in batches to the University of California San Francisco (UCSF) for mNGS on dry ice (Fig. 3).

Final clinical diagnoses and case classifications were adjudicated based on investigations performed in Uganda and per the consensus TBM UCD^8^ (Supplementary Table 4), respectively. Cases were categorized into definite TBM (microbiologically proven), probable TBM (score > 10 points), possible TBM (score 6–9 points), indeterminate (score < 6 points and without a microbiologically confirmed alternate infection), and Not-TBM (microbiologically confirmed alternate infection). Alternate diseases in the Not-TBM category included CM, bacterial meningitis, and viral meningitis (Table 1).

### Cerebrospinal fluid mNGS

Total nucleic acid was extracted from 90 uL of CSF using the Zymo *Quick*-DNA/RNA MagBead (Zymo Cat. No. R2130) via the Agilent Bravo or the Integra Viaflo 96, in batches of 40–96 samples and eluted into 50 uL of sterile water. The nucleic acid was then divided with half undergoing DNAse treatment to isolate RNA and the remainder being used for DNA sequencing (DNA-Seq). Total nucleic acid was also extracted from no-template water controls. ERCC RNAs were spiked into the RNA fractions at 25 pg to later back-calculate RNA mass and use as positive internal controls^23^.

RNA sequencing (RNA-Seq) libraries were prepared using the New England Biolabs’ NEBNext Ultra II RNA library preparation kit (NEB Cat No. E7770) as per the protocol. Library preparation was performed in bulk using the Echo Labcyte 525 and Agilent Bravo or Integra Viaflo 96 liquid handling robots^51^. DNA libraries were prepared using the New England Biolabs’ NEBNext® Ultra™ II FS DNA Library Prep Kit (E7805L) as per the protocol using the Echo Labcyte 525 and Agilent Bravo liquid handling robots. Host ribosomal RNA depletion was performed using the Qiagen QIAseq FastSelect RNA removal kit (Qiagen Cat No. 333180) at 1:100 dilution. The RNA-Seq libraries for 205 samples from both training and test cohorts underwent ERCC depletion using DASH. DASH is a CRISPR-Cas9 technology the removes abundant sequences from mNGS libraries. CRISPR-Cas-9 guide RNAs (gRNAs) were created to target ERCC sequences. DASH treatment is performed after ligation of adapters and unique barcoding of the RNA-seq library. gRNAs targe ERCC sequences. These regions in the library are cleaved, leaving only the fragments with intact adapters on both ends to be further amplified and sequenced^22^. Shallow sequencing was performed on an Illumina iSeq to calculate pooling volumes. Then pooled libraries were size selected using Ampure beads and sequenced on an Illumina Novaseq 6000 using 146 base pair paired-end sequencing.

### Metagenomic analysis

A open source, cloud-based metagenomics pipeline CZID was used for mNGS analysis^26^. Raw sequencing files are uploaded to CZID (czid.org), which performs several processing steps prior to analysis of non-host data. The first step in the pipeline is an alignment to the human genome to remove host sequences. Remaining sequences after this initial human alignment step were deposited in the National Center for Biotechnology Information (NCBI) Sequence Read Archive (SRA) with the primary accession codes PRJNA773920 (https://www.ncbi.nlm.nih.gov/bioproject/PRJNA773920). As a result, these data files contain non-host sequences not only from pathogens but also from environmental contaminants from skin and the laboratory, including bacteria, fungi, nonpathogenic viruses, and even vertebrates. Subsequent steps in the CZID pipeline (i.e., removal of low-quality reads, duplicate reads. low complexity reads, and additional human sequence filtering) further reduce contaminating and uninformative sequences from the nonhuman dataset. The remaining sequences undergo an assembly-based alignment using an indexed version of the NCBI’s GenBank database to identify the source of nonhuman sequences in the datasets. Additional computational steps detailed below that are specific to different types of pathogens were used to discriminate between likely environmental contaminants and candidate pathogens.

Given its paucibacillary nature, the abundance of TB DNA in CSF, as measured by rpM, can fall below the reporting thresholds in clinical mNGS assays^14^. We therefore identified the optimal reporting threshold for TB using the training cohort and used this threshold on the test cohort. Regarding common causes of bacterial meningitis, sequences aligning to *Streptococcus pneumoniae*, *Haemophilus influenzae*, and *Neisseria meningitidis* can be found in low abundance in CSF mNGS datasets secondary to environmental, specimen handling, and/or water contamination. Reads aligning to these pathogens were normalized using compression ratios obtained from the CZID pipeline. The relative abundance for these organisms was calculated in log10(rpM) by RNA-Seq (*x*-axis) versus DNA-Seq (*y*-axis) (Fig. 2C). When these bacteria were detected with both RNA and DNA abundance greater than 1 log relative to the mean abundance of the entire cohort, they were considered likely pathogens rather than environmental contaminants. A positive detection of a fungus or parasite required an rpM ratio of ≥10, where the rpM ratio = rpM CSF sample/rpM no-template control (NTC), or rpM ratio = rpM CSF sample if rpM NTC = 0^52^. Candidate viral pathogens had to have known neuroinvasive potential and at least one sequence and its mate pair both aligning specifically to the virus’ genome. Given that EBV and CMV can be incidentally detected by pathogen-specific PCR and/or CSF mNGS without clear disease association, only clear outlier cases defined as log10 (rPM) two-fold greater than the mean abundance of the entire cohort were presumed pathogenic within the context of patients with advanced HIV infection. Neuroinvasive viruses, bacteria, fungi, and parasites identified by mNGS were orthogonally validated with pathogen-specific PCR, clinical microbiological tests performed in Uganda (e.g., CrAg and fungal culture) or repeat mNGS on an independent CSF aliquot.

### Development of a host gene expression MLC to discriminate between TBM (without co-infection) and non-TB etiologies

We created a training cohort to identify the optimal reporting threshold for TB and to build the MLC classifier. The cohort included patients with definite TBM and other neurological diseases (ONDs). Patients from the OND group were selected from the probable TBM, possible TBM / indeterminate and Not-TBM groups, including cases with viral, bacterial, or fungal pathogens, along with cases with only low abundance HIV, EBV, and/or CMV. This was done to prevent biasing the training set to a particular OND and to provide a more accurate representation of the overall patient population.

The workflow for the training of the MLC is presented in Fig. 3A. Human gene counts were generated with Spliced Transcripts Alignment to a Reference (STAR) (v2.5.3a) from the CZID analysis pipeline^26^ using human genome assembly build 38 v23. Transcript counts for human protein-coding genes (*n* = 19,590) from the samples comprising the training set were used as an input for building the MLC. Samples were normalized using the trimmed mean of M-values using *calcNormFactors* and *logCPM* functions from the edgeR R package that were adapted in python v3.6.21^53^. Additionally, age, sex, GCS, and CSF white cell counts were included as a priori suspected co-variates. As the number of genes used as input (19,590) was larger than the number of samples used to train the classifier, we used feature selection (Univariate Feature Selection, scikit-learn v.0.21.3) to reduce the dimensionality of the input vector and identify the smallest set of genes most predictive of TBM and OND. Finally, five different ML methods (logistic regression, random forest, SVM, elastic net, and XGBoost) were evaluated to determine the best performing classifier. We chose to evaluate these MLC methods because they are “interpretable”. In other words, the genes used for classification are known so it is possible to extract decision rules. Final parameters for the MLC were chosen using cross-validation (80/20 split) to avoid overfitting. The classifier with the highest sensitivity (followed by AUC, and specificity) was chosen as the final classifier.

### Testing of a combined mNGS and host gene expression MLC

A test cohort of patients with TBM and ONDs was created to assess the sensitivity and specificity of the host gene expression MLC alone and the combined mNGS and host-based gene expression MLC assay for diagnosing patients with or without TBM. UCSF investigators were blinded to any sample or case details. The final combined assay used both the mNGS (both DNA-seq and RNA-seq) and host MLC resulting in a single test for the detection of TBM versus OND. If the MLC classified a case differently than the pathogens detected by the mNGS assay (meeting preset mNGS thresholds), then the mNGS results would overrule the MLC due to the presumption that direct detection of a pathogen was more specific than categorization of disease based on a host response signature. Patients with TBM for whom there was evidence of a CNS co-infection found on mNGS (other than HIV) were not used in the final test set. The reference standard was a microbiological composite of conventional CSF testing (e.g., CSF CrAg, fungal culture, Gram stain, bacterial culture, Gene Xpert MTB/RIF Ultra, TB culture) and orthogonally confirmed mNGS-identified pathogens (other than TB).

### In silico evaluation of mNGS and a host gene expression MLC with shallow depth sequencing for low resource settings

To assess whether similar results were achievable at shallow depth sequencing to better enable mNGS diagnostics in lower resource settings, we subsampled our test dataset at various sequencing depths (i.e., 100,000 to 2 million reads) and analyzed the impact on the sensitivity and specificity of the assay for pathogen identification and the accuracy of the host gene expression MLC. First, all samples were aligned to the ERCC transcripts using STAR (v2.5.3a) to filter out any remaining ERCC sequences that were not removed through DASH. Next, files were each subsampled in triplicate using *seqtk* (v1.2-r94) at the following sequencing depths: 100,000, 150,000, 200,000, 500,000, 1,000,000, and 2,000,000 reads. Subsampled files were then aligned to the human genome using STAR to obtain transcript counts, which were then run through MLC1 classifier. Subsampled files were also uploaded to the CZID pipeline for mNGS analysis.

### Statistics

Final diagnostic accuracy of the combined assay (AUC, sensitivity, and specificity) was based on comparing the results against a composite of clinical diagnostic testing results and orthogonally confirmed mNGS-identified pathogens. The *p*-value for the AUC was calculated in GraphPad Prism, using a two-tailed test for hypotheses and assuming that the null hypothesis value for the AUC was 0.5. Baseline clinical characteristics and demographic data were compared between cohorts via Mann-Whitney *U* for continuous variables and chi-square test for categorical variables.

### Reporting summary

Further information on research design is available in the Nature Research Reporting Summary linked to this article.

## Supplementary information

Supplementary Information

Description of Additional Supplementary Files

Supplementary Data 1

Supplementary Data 2

Reporting Summary

## Source data

Source Data

## Data Availability

Non-host sequence data that support the findings of this study have been deposited in NCBI Sequence Read Archive with the primary accession codes PRJNA773920. All host gene counts for samples have been deposited at https://github.com/UCSF-Wilson-Lab/TBM_classifier and Zenodo^54^. Source data are provided with this paper.

## Code availability

All code used in the generation of the machine learning classifier, and all gene counts used in the development of the machine learning classifier have been deposited at https://github.com/UCSF-Wilson-Lab/TBM_classifier and Zenodo^54^.
