# Author Correction: Integrating central nervous system metagenomics and host response for diagnosis of tuberculosis meningitis and its mimics

**DOI:** 10.1038/s41467-026-79080-w

**Published:** 2026-10-29

**Authors:** P. S. Ramachandran, A. Ramesh, F. V. Cresswell, A. Wapniarski, R. Narendra, C. M. Quinn, E. B. Tran, M. K. Rutakingirwa, A. S. Bangdiwala, E. Kagimu, K. T. Kandole, K. C. Zorn, L. Tugume, J. Kasibante, K. Ssebambulidde, M. Okirwoth, N. C. Bahr, A. Musubire, C. P. Skipper, C. Fouassier, A. Lyden, P. Serpa, G. Castaneda, S. Caldera, V. Ahyong, J. L. DeRisi, C. Langelier, E. D. Crawford, D. R. Boulware, D. B. Meya, M. R. Wilson

**Affiliations:** 1 Weill Institute for Neurosciences, Department of Neurology, University of California, San Francisco, San Francisco, CA USA; 2 University of Melbourne, Melbourne, VIC Australia; 3 UCSF Center for Tuberculosis, San Francisco, CA USA; 4 UCSF Center for Encephalitis and Meningitis, San Francisco, CA USA; 5 Clinical Research Department, London School of Hygiene and Tropical Medicine, London, UK; 6 Infectious Diseases Institute, Makerere University, Kampala, Uganda; 7 Medical Research Council—Uganda Virus Research Institute—LSHTM Uganda Research Unit, Entebbe, Uganda; 8 University of California School of Medicine, San Francisco, CA USA; 9 University of Minnesota, Minneapolis, MN USA; 10 Department of Biochemistry and Biophysics, University of California, San Francisco, San Francisco, CA USA; 11 Division of Infectious Diseases, Department of Medicine, University of Kansas, Kansas City, KS USA; 12 Chan Zuckerberg Biohub, San Francisco, CA USA; 13 Department of Medicine, University of California, San Francisco, San Francisco, CA USA

**Keywords:** Infectious-disease diagnostics, Pathogens, Meningitis, Tuberculosis

Correction to: *Nature Communications* https://doi.org/10.1038/s41467-022-29353-x, published online 30 March 2022

In the version of this article initially published, the surname of F. V. Cresswell was misspelled (Creswell) and is now amended in the HTML and PDF versions of the article.

