# Supplementary material for: Integrating central nervous system metagenomics and host response for diagnosis of tuberculosis meningitis and its mimics
Source: Nat Commun. 2022 Mar 30;13:1675. doi: 10.1038/s41467-022-29353-x (PMC8967864; doi:10.1038/s41467-022-29353-x)
Supplement: Supplementary file 1 — Supplementary Information [file 41467_2022_29353_MOESM1_ESM.pdf]

Supplementary Information

Integrating Central Nervous System Metagenomics and Host Response for Diagnosis of Tuberculosis Meningitis and Its Mimics

Ramachandran PS[1,2,3,4] *, Ramesh A[1,] *, Creswell FV[5,6,7], Wapniarski A[1], Narendra R[1], Quinn CM[8], Tran EB[8], Rutakingirwa MK[6], Bangdiwala AS[9], Kagimu E[6], Kandole KT[6], Zorn KC[4,10], Tugume L[6], Kasibante J[6], Ssebambulidde K[6], Okirwoth M[6], Bahr NC[11], Musubire A[6], Skipper CP[6,9], Fouassier C[1], Lyden A[12], Serpa P[12], Castaneda G[12], Caldera S[12], Ahyong V[12], DeRisi JL[4,10,12], Langelier C[12,13], Crawford ED[12], Boulware DR[9], Meya DB[6,9], Wilson MR[1,3,4]

1. Weill Institute for Neurosciences, Department of Neurology, University of California, San Francisco, San Francisco, CA, USA
2. University of Melbourne, Melbourne, VIC, Australia
3. UCSF Center for Tuberculosis, San Francisco, CA, USA
4. UCSF Center for Encephalitis and Meningitis, San Francisco, CA, USA
5. Clinical Research Department, London School of Hygiene and Tropical Medicine, London, UK
6. Infectious Diseases Institute, Makerere University, Kampala, Uganda
7. Medical Research Council - Uganda Virus Research Institute - LSHTM Uganda Research Unit, Entebbe, Uganda
8. University of California School of Medicine, San Francisco, CA, USA
9. University of Minnesota, Minneapolis, MN, USA
10. Department of Biochemistry and Biophysics, University of California, San Francisco, San Francisco, CA, USA
11. Division of Infectious Diseases, Department of Medicine, University of Kansas, Kansas City, KS, USA
12. Chan Zuckerberg Biohub, San Francisco, CA, USA

13. Department of Medicine, University of California, San Francisco, San Francisco, CA, USA

* Equal contribution

Table of Contents

***Supplementary Methods:* ... 2**

***Supplementary Note 1:* ... 2**

***Supplementary Note 2:* ... 4**

***Supplementary References:* ... 13**

***Supplementary Figure 1:* ... 14**

***Supplementary Figure 2:* ... 15**

***Supplementary Table 1:* ... 17**

***Supplementary Table 2:* ... 18**

***Supplementary Table 3:* ... 20**

***Supplementary Table 4:* ... 21**

***Supplementary Table 5:* ... 23**

Supplementary Methods:

For *de novo* assembly and annotation of Wesselsbron virus, we used the St. Petersburg genome assembler (SPAdes, v3.11.1) and Geneious (v10.3.2).[27] To build a phylogenetic tree of Wesselsbron virus, a multiple sequence alignment using the genome we assembled and reference genomes (obtained from NCBI) was built using MUSCLEv3.8.[28] The best-fitting evolutionary model was picked by ModelTest-NGv0.1.5, and a phylogenetic tree was built using RAxML-ng v0.6.0 (n=200 bootstraps).[29,30]

Supplementary Note 1:

As stated in the main Results section, 4 machine learning classifiers were run on the blinded test set: MLC1, MLC2, 4 gene and 7gene. In this section we discuss details and performance of the MLC2, 4 gene and 7 gene classifiers. MLC2, similar to MLC1, was a SVM classifier with L1 penalty, C = 0.1, prior count =75, and top 40 features, independent of the co-variate matrix, with the exception that MLC2 had a TB sample weight of 2, to further increase sensitivity. A cutoff of >50% was used for the diagnosis of TBM. MLC2 had an AUC = 0.93, sensitivity = 0.91 and specificity = 0.87 on the training cohort, and all the 10 re-sequenced samples were classified correctly. On the blinded test set, MLC2 had a sensitivity of 77.78% (CI 39.99-97.19%), specificity was 65.33% (CI 53.46-75.96%), and AUC of 0.74 (p=0.02). The combination of mNGS and MLC2 against microbiologically confirmed cases demonstrated a sensitivity, specificity and AUC of 88.89% (CI 51.75%-99.72%), 77.33% (CI 66.21-86.21%) and 0.85 (p=0.0006), respectively (Supplementary Table 5).

Based on the genes used predominantly (>500/1000 bootstrap) for classification of TBM, and TBM+OND, the 4 and the 7 gene classifiers were run on the blinded test set. A cutoff of >50% was used for the diagnosis of TBM. Both the 4 and 7 gene classifiers were SVM classifiers, with regularization parameter C = 0.1 and prior count = 75.

On the blinded test set, the 4 gene classifier had a sensitivity of 77.78% (CI 39.99-97.19%), specificity was 54.67% (CI 42.75-66.21%), and AUC of 0.74 (p=0.02). The combination of mNGS and 4 gene classifier against microbiologically confirmed cases demonstrated a sensitivity, specificity and AUC of 88.89% (CI 51.75%-99.72%), 70.67% (CI 59.02-80.62%) and 0.87 (p=0.0003), respectively (Supplementary Table 5).

On the blinded test set, the 7 gene classifier had a sensitivity of 77.78% (CI 39.99-97.19%), specificity was 66.67% (CI 54.83-77.14%), and AUC of 0.77 (p=0.007). The combination of

mNGS and 7 gene classifier against microbiologically confirmed cases demonstrated a sensitivity, specificity and AUC of 88.89% (CI 51.75%-99.72%), 78.67% (CI 67.68-87.29%) and 0.89 (p=0.0001), respectively (Supplementary Table 5).

To assess the power of our MLC, learning curves were generated for the best performing classifier: MLC1 (Supp fig 1). The training cohort was subsampled randomly at 50, 55, 60, 65, 70, 75, 80, 85, 90, 95 and 100% of total samples, and AUC and sensitivity were calculated for both the training and test cohorts. Each iteration was bootstrapped 1000 times. The mean test AUC and test sensitivity across all different sub-samples of training cohort (n=35 (50%) to n=66 (95%) samples) of training cohort was 0.76 (CI: 0.74-0.77) and 75.8% (CI:72.48-79.11%), comparable to the overall performance of MLC1 (test AUC = 0.74, test sensitivity = 77.78%).

Supplementary Note 2:

Case 1. A patient who was recently diagnosed with HIV and started on anti-retroviral therapy (ART) 3 months prior to presentation was admitted with subacute headache and fever progressing to altered mentation with one likely unobserved seizure. They were in respiratory distress and had nuchal rigidity, right upper limb hypertonia, and oral sores. Cerebrospinal fluid (CSF) was obtained with an opening pressure of 23 cmH_2O (normal: <20 cmH_2O) and with 45 leukocytes/µL (normal: <5 /µL) of which 100% were lymphocytes, 102 mg/dL protein (normal: 15-60 mg/dL), and 30 mg/dL glucose (normal: 50-80 mg/dL). Gene Xpert Ultra *Mycobacterium tuberculosis* (TB) PCR and TB culture of the CSF were negative. Cryptococcal antigen (CrAg) was positive in serum but negative in CSF. They were treated with fluconazole and amphotericin for symptomatic cryptococcal antigenemia, with ceftriaxone to cover for bacterial meningitis and pneumonia, and with rifampicin, isoniazid, pyrazinamide, ethambutol, and prednisone for possible TB meningitis (TBM). The patient continued spiking fevers up to 40ºC, developed a left lower limb deep vein thrombosis, and had respiratory deterioration requiring 5L oxygen. They died on hospital day 11. CSF mNGS detected 391.4 rPM (DNA) corresponding to

herpes simplex virus type 1 (HSV-1). With the clinical history of seizure, altered mentation, persistent fevers despite empiric treatment for TB, fungal and bacterial meningitis, and respiratory distress, the patient's presentation was consistent with HSV-1 meningoencephalitis and pneumonitis.

Original Case Classification: Possible TBM / indeterminate

Likely Final Diagnosis: HSV-1 meningoencephalitis

Case 2. A patient with HIV who discontinued ART one month prior presented with headache, fever, blurred vision and stiff neck, for which they received antimalarials without improvement. CSF was obtained with an opening pressure of 20 cmH_2O and showed 160 leukocytes /µL (100% lymphocytes), 58 mg/dL protein, 14 mg/dL glucose, and negative Gene Xpert Ultra. They were treated with ceftriaxone for suspected bacterial meningitis without improvement. A Biofire meningitis/encephalitis PCR panel was positive for HSV-2. They were treated with oral valacyclovir 1g three times daily and were discharged after showing improvement in symptoms. Consistent with the HSV-2 PCR results, CSF mNGS detected 189 rPM (DNA) corresponding to HSV-2.

Original Case Classification: HSV-2 meningitis (not-TBM group)

Likely Final Diagnosis: HSV-2 meningitis

Case 3. A patient with a history of pulmonary TB treated 5 months prior who presented with fever, altered mental status, seizures, neck stiffness, and hemiparesis. On exam they had signs of immunosuppression including oral thrush, significant wasting, and a rash characterized as a pruritic papular eruption. Their HIV PCR was positive, and their CD4 count was 10 cells/mm^3. CSF was obtained with an opening pressure of 9 cmH_2O, <5 leukocytes /µL, 23 mg/dL protein, and 31 mg/dL glucose. Serum and CSF CrAg were negative. CSF Gene Xpert Ultra was

negative, and CSF cultures for bacteria, fungi, and mycobacteria showed no growth. Their liver enzymes were elevated five times the upper limit of normal, felt to be a complication of past anti-TB therapy. The patient was empirically treated with ceftriaxone for bacterial meningitis with reported slight improvement. However, during the second week of hospitalization, the patient experienced a sudden decrease in consciousness and died; intracerebral hemorrhage was suspected, although no imaging could be performed. CSF mNGS detected 3799.7 rPM (DNA) corresponding to *Toxoplasma gondii* and 8.1 rPM (DNA) corresponding to HSV-2.

Original Case Classification: Possible TBM / indeterminate

Likely Final Diagnosis: CNS toxoplasmosis and HSV-2 meningoencephalitis

Case 4. A patient with HIV was referred from a rural hospital for difficulty feeding due to widespread oral ulcerations. They were finishing their second month of treatment for smear-positive pulmonary TB, during which time they had not taken ART due to past treatment failure. On admission, the caregiver reported 3 months of intermittent headache and depression that had progressed to fevers, photophobia, and lower limb weakness. They were found to be confused and to have meningismus with upper-motor-neuron pattern weakness and decreased sensation in their lower limbs with a sensory level. Their alertness was fluctuating, as were neurologic signs including generalized hyporeflexia, horizontal gaze palsy, nystagmus, and slurred speech. CSF was obtained with an opening pressure of 8 cm H_2O and was xanthochromic in appearance with <5 leukocytes/µL and 34.5mg/dL protein (glucose was unavailable); Gene Xpert Ultra and CrAg were negative on CSF. A spinal MRI was consistent with transverse myelitis at T3. Dexamethasone was added to their TB regimen for possible TB meningitis and myelitis, and they had slight improvement, restarting ART at day 12. However, they died suddenly two days later. CSF mNGS detected 4460.1 RPM (DNA) corresponding to VZV.

Original Case Classification: Transverse myelitis, (Possible TBM / indeterminate)

Likely Final Diagnosis: VZV meningoencephalomyelitis

Case 8. A patient with HIV on ART for 1 year and a history of pulmonary TB treated 6 months prior to presentation, presented with a week of headache, neck stiffness, and rash. Exam showed a dermatomal thoracic rash. CSF was obtained with unmeasured opening pressure and showed 260 leukocytes/µL (100% lymphocytes), 177 mg/dL protein, 36 mg/dL glucose, and 4.5 mmol/L lactate (normal: <3 mmol/L). Gene Xpert Ultra was negative, and a Biofire meningitis / encephalitis multiplex PCR was positive for VZV. They were treated with oral acyclovir with rapid improvement and was discharged. CSF mNGS detected 5.1 rPM (DNA) corresponding to VZV.

Original Case Classification: VZV meningitis, (not-TBM group)

Likely Final Diagnosis: VZV meningitis

Case 10. A patient with HIV on ART for nine years presented with one month of headache, confusion, and blurred vision. On exam, they were disoriented, their neck was supple, and they had monocular vision impairment. CSF was obtained with an opening pressure of 18 cmH_2O, and with <5 leukocytes/µL, 99 mg/dL protein, 38 mg/dL glucose, and 4.6 mmol/L lactate. CSF Gene Xpert Ultra and CrAg were negative. The patient was initially treated with doxycycline and ceftriaxone. Serum cytomegalovirus (CMV) IgG later returned positive. They were discharged after their headache and confusion improved. CSF mNGS detected 5607.5 rPM (DNA) corresponding to CMV, which was confirmed by PCR.

Original Case Classification: Possible CMV encephalitis (Possible TBM / indeterminate

Likely Final Diagnosis: Possible CMV encephalitis

Case 12. A patient presented two months after being diagnosed with HIV and initiated on ART. They complained of headache and new onset of paranoid delusions and aggressive behavior,

which progressed to severe weakness and inability to walk in the days before presentation. On exam, they were ill-appearing and confused, but had a supple neck and no focal motor deficits. Purple Kaposi sarcoma-like lesions were noted on the oral mucosa. CSF was obtained with unmeasured opening pressure, <5 leukocytes/µL 53 mg/dL protein, glucose (44 mg/dL) less than half the serum glucose, and 2.9 mmol/L lactate. CSF CrAg and Gene Xpert Ultra were negative. CD4 count on admission was 45 cells/mm^3; the value at HIV diagnosis was unknown. A non-contrast head CT scan showed only diffuse cerebral atrophy. The patient was presumed to have TB meningoencephalitis based on the low CSF glucose, and was started on four-drug TB therapy including high dose oral rifampin (35 mg/kg) and dexamethasone after enrollment in a clinical trial. The patient had some improvement in alertness but remained confused. There were discharged at week 3 to receive outpatient chemotherapy for their oral Kaposi sarcoma which had been confirmed pathologically. They remained cognitively impaired and died five months later while still receiving chemotherapy. CSF mNGS detected 377.9 rPM (DNA) corresponding to CMV.

Original Case Classification: Possible TBM / indeterminate

Likely Final Diagnosis: CMV encephalitis

Case 14. A patient with HIV not on ART presented with 2 months of headache and blurry vision. One year prior they were diagnosed with cryptococcal meningitis (CM) and treated with 14 days of amphotericin, but was lost to follow up and never received fluconazole prophylaxis or ART. On exam they had a stiff neck, and significant pallor. A CBC was notable for hemoglobin of 6 g/dL (13.5-17.5g/dL), and their serum CrAg was positive. CSF was obtained with an opening pressure of 18 cmH_2O, positive CrAg, <5 leukocytes, 60 mg/dL protein,116 mg/dL glucose, 3.1 mmol/L lactate. CSF smear and fungal culture were negative. The patient was treated for CM relapse with fluconazole and amphotericin, and was discharged on day 12, before negative

cryptococcal CSF cultures were noted. CSF mNGS detected 78.5 rPM corresponding to human parvovirus B19.

Original Case Classification: Cryptococcal meningitis relapse, (not-TBM group)

Likely Final Diagnosis: Human parvovirus B19 meningitis

Case 15. A patient with HIV on ART presented to an outside hospital with chronic headache and cough, associated with fever, B symptoms, and neck stiffness. At that hospital, their CSF profile showed <5 leukocytes/µL, 29.1 mg/dL protein, and 55 mg/dL glucose, with a positive CSF CrAg. They were treated for CM and received 2 days of amphotericin and fluconazole before transfer. At Kiruddu National Referral Hospital, a repeat lumbar puncture showed an opening pressure of 20 cmH_2O, and positive CSF cryptococcal culture. There was initial clinical improvement on amphotericin, but after a week they had become febrile (38.8ºC) and was given doxycycline for phlebitis. Fevers persisted, and they developed hypoxia, pallor, and returned neck stiffness. Hepatosplenomegaly and bilateral pleural effusions were seen on ultrasound. A CBC showed hemoglobin 2.8 g/dL (13.9 g/dL on admission) with stable thrombocytopenia of 138,000 platelets/µL (normal: 150,000-450,000 /µL), and they were transfused a total two units of whole blood. They were treated for fever of unknown origin with piperacillin/tazobactam but fevers up to 39.3ºC persisted for ten days. Despite a negative malaria rapid diagnostic test, a trial of coartem was given, after which their fevers were low grade. They were discharged on day 15 still ill and with intermittent low-grade fevers, but with improved respiratory status and normal mental status. The patient was lost to follow-up, and subsequent attempts to contact the family were unsuccessful. CSF mNGS detected 6663 rPM corresponding to measles virus, as well as 1571 rPM to *Cryptococcus neoformans*. Notably, this patient presented in July 2018, the peak of a three-year measles outbreak in Uganda.[1]

Original Case Classification: CM complicated by persistent fever, (not-TBM group)

Likely Final Diagnosis: CM and measles virus co-infection causing possible measles inclusion body encephalitis.[2]

Case 16. A patient with a history of pulmonary TB and advanced HIV who initiated ART three weeks prior to admission presented with altered mentation, vomiting, weight loss, and cough. They were tachypneic, had severe temporal wasting, abdominal tenderness, hepatosplenomegaly, and meningismus. CSF was obtained with an opening pressure of 13 cmH_2O, and analysis showed <5 leukocytes/µL, 18 mg/dL protein, 58 mg/dL glucose, and 7 mmol/L lactate, with a negative CSF CrAg despite their positive serum CrAg. CSF Gene Xpert Ultra was negative, and fungal and mycobacterial cultures had no growth. They developed respiratory distress with a high fever and a chest x-ray showing widespread reticular opacities. An abdominal ultrasound showed splenic abscesses and widespread abdominal lymphadenopathy consistent with abdominal TB. They initially received fluconazole monotherapy for CM without improvement, and then four-drug antitubercular therapy with prednisone for disseminated TB and possible TBM. The patient died on day four of admission. CSF mNGS detected 23.4 rPM corresponding to TB (DNA) and 5739.5 rPM (RNA) corresponding to Wesselsbron virus, a positive-sense RNA arbovirus of the flavivirus genus prevalent in eastern and southern Africa[3]. There is one previous report of Wesselsbron virus encephalitis resulting from a laboratory accident[4]. While there are 41 reported human cases, typically of an influenza-like illness with hepatomegaly[5,6], it has never been reported in an HIV-positive patient, and therefore this severe presentation could represent Wesselsbron meningoencephalitis.

Original Case Classification: Possible TBM / indeterminate

Likely Final Diagnosis: TBM and Wesselsbron meningoencephalitis

Case 23. A patient with HIV (ART naïve with most recent CD4 count of 9 cells/ mm^3) presented with two weeks of headache and B symptoms, progressing to confusion. On exam, they were cachectic, their neck was stiff, they had unilateral facial weakness, and was not localizing to painful stimulus (Glasgow coma scale (GCS) = 13). CSF was obtained with an opening pressure of 23 cmH_2O, <5 leukocytes/µL, 131 mg/dL protein, 76 mg/dL glucose, and 5.2 mmol/L lactate. Gene Xpert Ultra was positive on CSF and urine, and they were started on four-drug anti-TB therapy for TBM as well as dexamethasone. The patient initially worsened with new hemiparesis, and decreasing GCS to 11, before their neurologic status improved by day 10. Their course was complicated by hyponatremia, aspiration pneumonia, persistent fevers, and drug-induced liver injury. After three weeks of hospitalization, the patient was well-enough for discharge, and continued improving through 24 weeks of follow-up. CSF mNGS detected 2305.4 rPM corresponding to *T. gondii*, as well as 0.1 rPM corresponding to TB.

Original Case Classification: Definite TBM

Likely Final Diagnosis: TBM and CNS toxoplasmosis

Case 24. A patient presented with 2 weeks of headache and vomiting. Their exam was notable only for oral thrush; there were no focal neurologic deficits. They were found to be HIV-positive and was initiated on ART; their CD4 count was 23 cells/ mm^3. CSF was obtained with an opening pressure of 35 cmH_2O and analysis showed 40 lymphocytes/µL (100% lymphocytes), 174 mg/dL protein, 41 mg/dL glucose, and 3.9 mmol/L lactate. CrAg was negative on CSF and serum, and CSF Gene Xpert Ultra was negative. The patient was treated with antitubercular therapy and dexamethasone for presumed TBM. They improved rapidly on treatment, with full resolution by week 4. CSF mNGS detected 242.4 rPM (DNA) corresponding to *T. gondii*, and 50.3 rPM (RNA) corresponding to *C. neoformans*.

Original Case Classification: Probable TB Meningitis

Likely Final Diagnosis: Possible Meningoencephalitis due to toxoplasma and cryptococcal coinfection

Case 25. A patient was diagnosed with HIV upon presentation to the emergency department with subacute headache, photophobia, and altered mental status. On exam, the patient was confused, but without neck stiffness or focal neurologic deficit. CSF was obtained with unmeasured but subjectively elevated opening pressure and 145 leukocytes /µL (100% lymphocytes); protein and glucose were unmeasured. CrAg was positive in serum and CSF. Antifungals were initiated for presumed CM, although CSF fungal cultures eventually resulted as negative. The patient's condition continued to deteriorate with decreasing level of consciousness and development of anisocoria, and the patient died during the hospitalization. CSF mNGS detected 653.7 rPM (DNA)corresponding to *T. gondii*.

Original Case Classification: Cryptococcal meningitis, (not-TBM)

Likely Final Diagnosis: CNS toxoplasmosis

Case 39. A patient with HIV presented with one week of headache progressing to neck stiffness and seizures. They had been taking ART for 2 years but switched to a dolutegravir-based regimen one month prior to presentation after diagnosis of herpes zoster. CSF was obtained with an opening pressure of 23.5 cmH_2O and showed 5,040 leukocytes/µL (87% lymphocytes), 216 mg/dL protein, and 29 mg/dL glucose. CSF Gene Xpert Ultra was negative, but the patient was started on four-drug antitubercular therapy for suspected TBM. The patient had a decrease in level of consciousness over the next three days, now with a GCS score of 11, when a Biofire M/E PCR panel returned positive for *Neisseria meningitidis*. After initiating antibiotics, the patient improved dramatically, returning to work as a construction worker by 3 months after presentation. CSF mNGS detected 1964.1 rPM on DNA and 22646.3 rPM on RNA corresponding to *N. meningitidis.*

Original Case Classification: Meningococcal meningitis, (not-TBM group)

Likely Final Diagnosis: Meningococcal meningitis

Supplementary References:

1. World Health Organization. Monthly Measles Surveillance Data. WHO. July 2019
2. Wilson M, Ludlow M, Duprex WP. (2013). Human Paramyxoviruses and Infections of the Central Nervous System. 10.1201/b13908-17.
3. Weiss KE, Haig DA, Alexander RA. Wesselsbron Virus – A virus not previously described, associated with abortion in domestiv animals. Onderstepoort Journal of Veterinary Research. 1956 Oct. 27:2:183-195
4. Weinbren MP. Some clinical observations on a human case of infection with Wesselsbron virus. East African Virus Research Institute Report July 1958– June 1959, 1959:22
5. Diagne MM, Faye M, Faye O, Sow A, Balique F, Sembène M, Granjon L, Handschumacher P, Faye O, Diallo M, Sall AA. Emergence of Wesselsbron virus among black rat and humans in Eastern Senegal in 2013. One Health. 2017 Feb 9;3:23-28. doi: 10.1016/j.onehlt.2017.02.001.
6. Weyer J., Thomas J., Leman P.A., Grobbelaar A.A., Kemp A., Paweska J.T. Human cases of Wesselsbron disease, South Africa 2010–2011. Vector Borne Zoonotic Dis. 2013 May;13(5):330–336

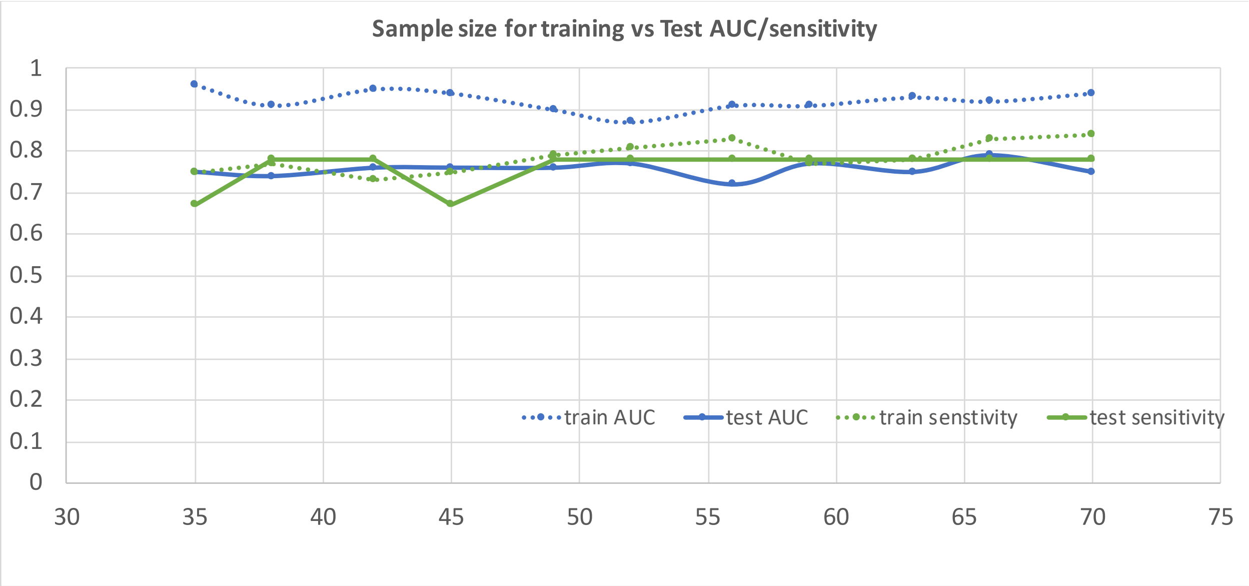

Supplementary Figure 1: Learning curves for training and test cohort, plotted with respect to Area Under Curve (AUC) and sensitivity.

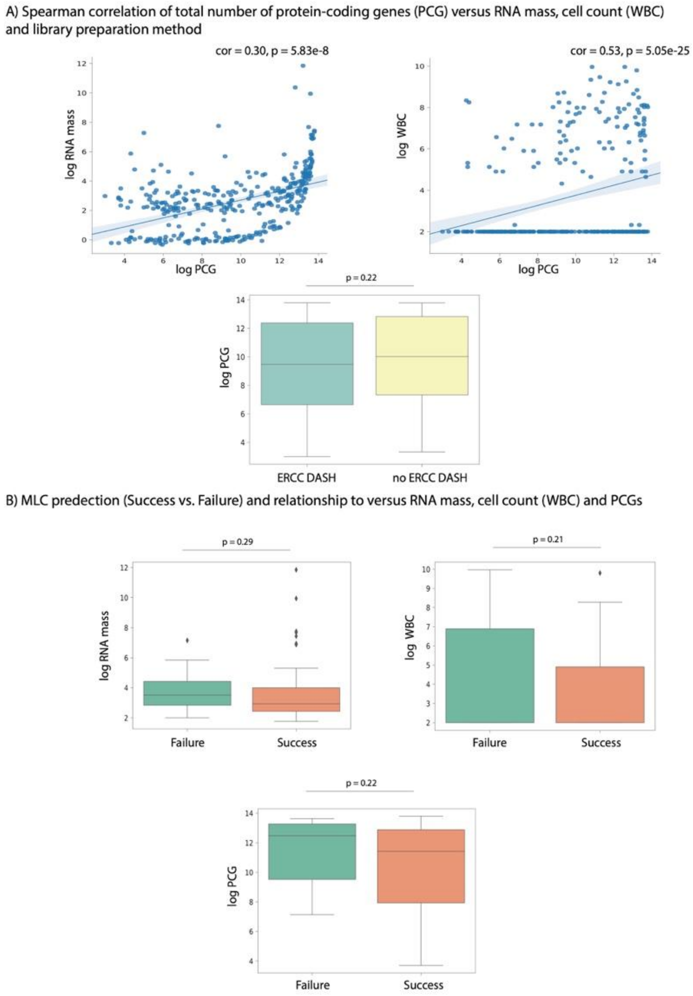

Supplementary Figure 2:

A) Spearman Correlation between total number of protein-coding genes (n=339 biologically independent samples, examined over 4 independent experiments) in each sample versus RNA mass (n=339), cell count (n=315) and library preparation method: ERCC DASH (n=191) and no ERCC DASH (n=148), represented as box/whisker plots. The relationship between protein-coding genes versus library preparation methods are represented as box/whisker plots, and no significant difference was observed using Wilcoxon rank sum test.

B) MLC1 success (correct prediction) and failures (incorrect prediction) on blinded test set (n=82 biologically independent samples, examined over 2 independent experiments), and relationship to cell count (n=79), total number of protein-coding genes in each sample (n=82), and RNA mass (n=81). There was no significant difference between the comparisons; significance was determined using Wilcoxon rank sum test. RNA, ribonucleic acid; WBC, white blood cells; PCG, protein coding genes; MLC, machine learning classifier; ERCC, External RNA Controls Consortium; DASH, Depletion of Abundant Sequences by Hybridization.

Baseline Demographics by Model Cohort							
	**Overall**		**Training Cohort**		**Test Cohort**		
No. participants	366		238		128		
**Demographics**	**N**	**Median [IQR] or N (%)**	**N**	**Median [IQR] or N (%)**	**N**	**Median [IQR] or N (%)**	**p-value**
Age (years)	366	35 [28, 41]	238	35 [29, 42]	128	35 [28, 40]	0.42
Female	366	145 (42.3%)	238	102 (42.9%)	128	54 (42.2%)	0.90
Headache duration, days	310	14 [7, 21]	204	14 [7, 21]	106	14 [7, 21]	0.45
**Clinical**	**N**	**Median [IQR] or N (%)**	**N**	**Median [IQR] or N (%)**	**N**	**Median [IQR] or N (%)**	**p-value**
Alive at discharge or last contact	361	267 (74%)	237	172 (72.6%)	124	95 (76.6%)	0.41
Microbiologically confirmed TBM (Definite TBM)	38	38 (10.4%)	30	30 (12.6%)	8	8 (6.3%)	
Microbiologically negative TBM but high clinical suspicion (probable TBM)	23	23 (6.3%)	10	10 (4.2%)	13	13 (10.2%)	
Microbiologically positive non-TBM (CM, ABM, VM)	201	201 (54.9%)	129	129 (54.2%)	72	72(56.3%)	
Possible/Indeterminate TBM	104	104 (28.4%)	69	69 (29.0%)	35	35 (27.3%)	

Supplementary Table 1: Baseline characteristics of training and test cohorts. Kruskal-Wallis test to compare medians; chi-square test for proportions. 2 participants were in both cohorts and were thus excluded from this table. TBM, tuberculous meningitis; CM, cryptococcal meningitis; ABM, acute bacterial meningitis; VM, viral meningitis

Patient Number	Positive on DNA-seq	Positive on RNA-seq
16	+	
22_CSF_06_27_19	+	
53		+
56	+	
61	+	+
64	+	
70	+	
102	+	
103	+	+
127	+	
133	+	
140	+	+
145	+	
153	+	+
155	+	
164	+	
171	+	
190	+	+
201	+	
204_CSF_repeat	+	
209	+	
211_CSF_01_14_19	+	
216	+	
218	+	
219_CSF_02_14_19	+	
224	+	
226	+	
234	+	+
237	+	
241	+	+
247	+	
261		+
284		+
309	+	
313	+	
319	+	
225_CSF_02_04_19	+	
225_CSF_03_04_19	+	+

Supplementary Table 2: Samples positive for TB through DNA and RNA-seq. DNA, Deoxyribonucleic acid; RNA, Ribonucleic acid

Patient Number	DNA-seq	2000000 reads	1000000 reads	500000 reads	200000 reads	150000 reads	100000 reads
34	Toxoplasma	+	+	+	+	+	+
33	Toxoplasma	+	+	+	+	+	+
30	Toxoplasma	+	+	+	+	+	+
29	Toxoplasma	+	+	+	+	+	+
31	Toxoplasma	+	+	+	+	+	+
6	Toxoplasma	+	+	+	-	-	-
23	Toxoplasma	+	+	+	+	+	+
6	VZV	+	+	+	+	+	+
275	VZV	+	+	+	-	-	-
12	CMV	+	+	+	+	+	+
18	EBV	+	+	+	+	+	+
19	EBV	+	+	+	+	+	+
38	Neisseria Meningitidis	+	+	+	+	+	+
41	Neisseria Meningitidis	+	+	+	+	+	+
39	Neisseria Meningitidis	+	+	+	+	+	+
56	TB	+	+	+	-	-	-
70	TB	+	+	+	+	+	-
102	TB	+	+	+	+	+	-
103	TB	+	-	-	-	-	-
16	TB	+	-	-	-	-	-
133	TB	+	+	+	+	+	+
190	TB	+	+	+	+	+	+
201	TB	+	-	-	-	-	-
219_CSF_02_14_19	TB	+	-	-	-	-	-
61	TB	+	+	+	-	-	-
225_CSF_02_04_19	TB	+	+	+	-	-	-
234	TB	+	+	+	+	+	+
241	TB	+	-	-	-	-	-
237	TB	+	-	-	-	-	-
64	TB	-	-	-	-	-	-
127	TB	-	-	-	-	-	-
164	TB	-	-	-	-	-	-
171	TB	-	-	-	-	-	-
216	TB	-	-	-	-	-	-
218	TB	-	-	-	-	-	-
224	TB	-	-	-	-	-	-
226	TB	-	-	-	-	-	-
247	TB	-	-	-	-	-	-
313	TB	-	-	-	-	-	-

319	TB	-	-	-	-	-	-
	**RNA-seq**						
38	Neisseria Meningitidis	+	+	+	+	+	+
40	Neisseria Meningitidis	+	+	+	+	+	+
41	Neisseria Meningitidis	+	+	+	+	+	+
39	Neisseria Meningitidis	+	+	+	+	+	+
6	Toxoplasma	+	+	+	+	+	+
6	VZV	+	+	+	+	+	+
103	TB	+	+	+	+	+	+
140	TB	+	+	+	+	+	+
153	TB	+	+	+	+	+	+
225_CSF_03_04_19	TB	+	+	+	+	+	+
234	TB	+	+	+	+	+	+
241	TB	+	+	+	+	+	+
61	TB	+	+	+	+	+	+
284	TB	+	+	+	+	+	+
190	TB	+	+	+	+	+	-

Supplementary Table 3: Decreasing subsampling depths and results of detection for each pathogen on RNA-seq and DNA-seq. Each sample was subsampled in triplicate with a different seed at varying sequencing depths. Positive result if all triplicate samples were positive. TB, Mycobacterium tuberculosis; VZV, varicella zoster virus; CMV, cytomegalovirus; EBV, Epstein-Barr virus.

**Uniform Case Definition**		
Clinical Criteria (maximum category score = 6)		Score
	Symptom Duration >5 days	4
	Systemic symptoms suggestive of TB (1 or more: weight loss/poor weight gain in children, night sweats, persistent cough greater than 2 weeks)	2
	History of recent close contact with an individual with pulmonary TB or a positive TST/IGRA in a child aged <10y	2
	Focal neurological deficit (excluding cranial nerve palsies)	1
	Cranial nerve palsy	1
CSF criteria (maximum category score = 4)	Clear appearance	1
	Cells: 10-500/ul	1
	Lymphocytic predominance (>50%)	1
	Protein concentration >1g/L	1
	CSF:plasma glucose ratio of <50% or an absolute CSF glucose concentration <2.2 mmol/L	1
Cerebral imaging (maximum category score = 6)	Hydrocephalus (CT and/or MRI)	1
	Basal meningeal enhancement (CT and/or MRI)	2
	Tuberculoma (CT and/or MRI)	2
	Infarct (CT and/or MRI)	1
	Precontrast basal hyperdensity (CT)	2
Evidence of Tuberculosis elsewhere (maximum category score = 4)	Chest radiograph suggestive of active tuberculosis (excludes miliary TB)	2
	Chest radiograph suggestive of miliary TB	4
	CT/MRI/US evidence of tuberculosis outside of the CNS	2
	AFB identified or *Mycobacterium tuberculosis* cultured from another site	4
Exclusion of an alternative diagnoses: an alternative diagnosis must be confirmed microbiologically, serologically or histologically		
Classification (Radiology not available / available)		
Definite TBM	Microbiologically proven	
Probable TBM	Diagnostic score > 10/12	
Possible TBM	Diagnostic score 6-9 / 6-11	
Indeterminate	Diagnostic score < 6	
Not-TBM	Microbiologically proven alternate cause identified	

Supplementary Table 4: Tuberculous Meningitis Uniform Case Definition. Scoring system consisting of clinical, laboratory and radiological (if available) criteria. TBM, Tuberculous meningitis; TST, tuberculin

skin test; IGRA, interferon gamma release assay; CSF, cerebrospinal fluid; CT, computer tomography; MRI,
magnetic resonance imaging; US, ultrasound; CNS, central nervous system; AFB, acid fast bacil

	AUC	p-value	Sensitivity (95% CI)	Specificity (95% CI)
**7 gene classifier**	0.774 (0.592-0.957)	0.0075	77.8 (40.0-97.2%)	66.7 (54.8- 77.1%)
**4 gene classifier**	0.743 (0.548-0.938)	0.0177	77.8 (40.0-97.2%)	54.7 (42.8-66.2%)
**MLC1**	0.739 (0.502-0.977)	0.0195	77.8 (40.0-97.2%)	76.0 (64.8-85.1%)
**MLC2**	0.739 (0.513-0.966)	0.0195	77.8 (40.0-97.2%)	65.3 (53.5-76.0%)
**7 gene + mNGS**	0.890 (0.777-1.00)	0.0001	88.9 (51.8-99.7%)	78.7 (67.7-87.3%)
**4 gene + mNGS**	0.873 (0.763-0.984)	0.0003	88.9 (51.8-99.7%)	70.7 (59.0-80.6%)
**MLC1 + mNGS**	0.872 (0.714-1.03)	0.0003	88.9 (51.8-99.7%)	86.7 (76.8-93.4%)
**MLC2 + mNGS**	0.851 (0.676 to 1.03)	0.0006	88.9 (51.8-99.7%)	77.3 (66.2-86.2%)

Supplementary Table 5: Results for individual MLC classifiers and in combination with mNGS pathogen detection results. AUC, area under the receiver operator characteristics curve, CI, confidence interval, MLC, machine learning classifier; mNGS, metagenomic next generation sequencing.
