# Supplementary material for: Integrating central nervous system metagenomics and host response for diagnosis of tuberculosis meningitis and its mimics
Source: Nat Commun. 2022 Mar 30;13:1675. doi: 10.1038/s41467-022-29353-x (PMC8967864; doi:10.1038/s41467-022-29353-x)
Supplement: Supplementary file 2 — Description of Additional Supplementary Files [file 41467_2022_29353_MOESM2_ESM.pdf]

Description of Additional Supplementary Files

File Name: Supplementary Data 1
Description:

a. List of all genes and the number of times they were used during the bootstrap process (n=1,000) for prediction of TB vs Other Neurologic Infection (ONI) for MLC1
b. List of all genes and the number of times they were used during the bootstrap process (n=1,000) for prediction of TB vs ONI for MLC2
c. List of genes used to for prediction of TB vs ONI in the 7 gene and 4 gene classifier

File Name: Supplementary Data 2
Description: List of all additional viruses found in entire cohort. These were found to be in low abundance in most cases and not felt to be pathogenic. Due to limitation with local radiology access, it was difficult to discern whether HIV was causative in most cases despite very high abundance on CSF. rPM, reads per million
