# Supplementary material for: Integrating central nervous system metagenomics and host response for diagnosis of tuberculosis meningitis and its mimics
Source: Nat Commun. 2022 Mar 30;13:1675. doi: 10.1038/s41467-022-29353-x (PMC8967864; doi:10.1038/s41467-022-29353-x)
Supplement: Supplementary file 5 — Reporting Summary [file 41467_2022_29353_MOESM5_ESM.pdf]

nature portfolio

Corresponding author(s): Michael Wilson, MD

Last updated by author(s): Mar 2, 2022

Reporting Summary

Nature Portfolio wishes to improve the reproducibility of the work that we publish. This form provides structure for consistency and transparency in reporting. For further information on Nature Portfolio policies, see our Editorial Policies and the Editorial Policy Checklist.

Statistics

For all statistical analyses, confirm that the following items are present in the figure legend, table legend, main text, or Methods section.

n/a	Confirmed	
[ ]	[x]	The exact sample size (*n*) for each experimental group/condition, given as a discrete number and unit of measurement
[ ]	[x]	A statement on whether measurements were taken from distinct samples or whether the same sample was measured repeatedly
[ ]	[x]	The statistical test(s) used AND whether they are one- or two-sided<br>*Only common tests should be described solely by name; describe more complex techniques in the Methods section.*
[ ]	[x]	A description of all covariates tested
[ ]	[x]	A description of any assumptions or corrections, such as tests of normality and adjustment for multiple comparisons
[ ]	[x]	A full description of the statistical parameters including central tendency (e.g. means) or other basic estimates (e.g. regression coefficient) AND variation (e.g. standard deviation) or associated estimates of uncertainty (e.g. confidence intervals)
[ ]	[x]	For null hypothesis testing, the test statistic (e.g. *F*, *t*, *r*) with confidence intervals, effect sizes, degrees of freedom and *P* value noted<br>*Give P values as exact values whenever suitable.*
[x]	[ ]	For Bayesian analysis, information on the choice of priors and Markov chain Monte Carlo settings
[x]	[ ]	For hierarchical and complex designs, identification of the appropriate level for tests and full reporting of outcomes
[x]	[ ]	Estimates of effect sizes (e.g. Cohen's *d*, Pearson's *r*), indicating how they were calculated

Our web collection on statistics for biologists contains articles on many of the points above.

Software and code

Policy information about availability of computer code

Data collection	We prospectively collected clinical data using paper case report forms (CRFs) digitally scanned into an intelligent character recognition system (Datafax 2018, version 5.1.4) with then further human error checking performed. We appreciate the support of the NIH NIAID Office of Cyber Infrastructure and Computational Biology.
Data analysis	Sequencing data was processed through CZID.org for metagenomics. Human gene counts were generated with Spliced Transcripts Alignment to a Reference (STAR) (v2.5.3a) from the CZID pipeline using human genome assembly build 38 v23. Samples were normalized using the trimmed mean of M-values using calcNormFactors and logCPM functions from the edgeR R package that were adapted in python v3.6.21.27. As the number of genes used as input (19,590) was larger than the number of samples used to train the classifier, we used feature selection (Univariate Feature Selection, scikit-learn v.0.21.3) to reduce the dimensionality of the input vector and identify the smallest set of genes most predictive of TBM and OND. The script has been deposited at https://github.com/UCSF-Wilson-Lab/TB-meningitis-CSF-classifier. In Silico subsampling was performed using STAR (v2.5.3a) and seqtk (v1.2-r94). We used Excel v16.54 for storage of final clinical and sequencing data and scatter plot generation. We used We used GraphPad Prism 7 for generation of ROC curves. We used Biorender.com to generate additional figures.

For manuscripts utilizing custom algorithms or software that are central to the research but not yet described in published literature, software must be made available to editors and reviewers. We strongly encourage code deposition in a community repository (e.g. GitHub). See the Nature Portfolio guidelines for submitting code & software for further information.

Data

Policy information about availability of data

All manuscripts must include a data availability statement. This statement should provide the following information, where applicable:

- Accession codes, unique identifiers, or web links for publicly available datasets
- A description of any restrictions on data availability
- For clinical datasets or third party data, please ensure that the statement adheres to our policy

All host transcriptomic gene counts generated through the study and used for the MLC was deposited at https://github.com/UCSF-Wilson-Lab/TB-meningitis-CSF-classifier (DOI:10.5281/zenodo.6207665). The non-human sequence reads from each sample were deposited at the National Center for Biotechnology Information Sequence Read Archive (PRJNA773920)

Field-specific reporting

Please select the one below that is the best fit for your research. If you are not sure, read the appropriate sections before making your selection.

☒ Life sciences ☐ Behavioural & social sciences ☐ Ecological, evolutionary & environmental sciences

For a reference copy of the document with all sections, see nature.com/documents/nr-reporting-summary-flat.pdf

Life sciences study design

All studies must disclose on these points even when the disclosure is negative.

Sample size	We did not perform a power calculation. We enrolled all eligible patients who presented over a 2 year period.
Data exclusions	There were no specific exclusion criteria. Patients had to be HIV positive and present with a clinical syndrome consistent with meningitis to be enrolled in the study. 1 CSF sample was excluded from the study because it was mislabeled at the recruitment site.
Replication	All mNGS results were replicated and confirmed. CSF was re-extracted and pathogens were confirmed using either PCR or repeat mNGS.
Randomization	Samples were not randomized into the training and test cohort. The first 240 patients who were recruited and had CSF sequencing were allocated to the training group. The remainder of patients after this time period were allocated into the test group. Sup Table 2 demonstrates the baseline characteristics of the two groups. We found no differences in demographics or presentation between the two groups and we did not perform any further statistical measures for control of co-variates between the groups though co-variates were controlled for in the development of the machine learning classifier.
Blinding	Once the training cohort was recruited and sequenced, UCSF researchers were then blinded to all clinical information of patients who were recruited and allocated to the test cohort. Samples were shipped to UCSF and these underwent sequencing and data analysis. Once final results for the test cohort were determined, the clinical data was unlocked and provided to the UCSF researchers to assess the sensitivity and specificity of the assay against the current gold standard.

Reporting for specific materials, systems and methods

We require information from authors about some types of materials, experimental systems and methods used in many studies. Here, indicate whether each material, system or method listed is relevant to your study. If you are not sure if a list item applies to your research, read the appropriate section before selecting a response.

Materials & experimental systems

n/a	Involved in the study	
☒	☐	Antibodies
☒	☐	Eukaryotic cell lines
☒	☐	Palaeontology and archaeology
☒	☐	Animals and other organisms
☐	☒	Human research participants
☐	☒	Clinical data
☒	☐	Dual use research of concern

Methods

n/a	Involved in the study	
☒	☐	ChIP-seq
☒	☐	Flow cytometry
☒	☐	MRI-based neuroimaging

Human research participants

Policy information about studies involving human research participants

Population characteristics	The median age was 35 years [IQR 29-41years] with 42.7% female. All patients were HIV positive. Amongst the patients for whom the CD4 cell count was measured (n=95), the median was 41 cells/mm3 [IQR 13-80cells/mm3]. At the time of admission, 181 patients (49.1%) were documented as being on antiretroviral therapy (ART), with 41/181 (22.6%) of these patients known to have commenced ART in the prior month. Median Glasgow Coma Scale at presentation was14 [IQR 14-15], 185/362 (51.1% of those with known GCS) had GCS < 15.
Recruitment	Patients were prospectively recruited as part of the “Improving Diagnostics and Neurocognitive Outcomes in HIV/AIDS-related Meningitis" study (NCT01802385) (IRB MREC1246; UMN1304M31361), a prospective cohort study underway in Uganda. All HIV-positive patients presenting with signs and symptoms concerning for meningitis (some combination of headache, fever, nuchal rigidity, neurologic deficit, or altered mental status) to Kiruddu Regional Referral Hospital, Kampala, Uganda from March 2018 to March 2020 were screened for study inclusion. If patients met the clinical criteria for inclusion and had CSF, then they were recruited into our study. Therefore we do not believe there was significant local selection bias, but the study results may not be generalizable beyond HIV-infected, Ugandan adults.
Ethics oversight	Research was approved by University of Minnesota (IRB Study ID STUDY00006856), Infectious Diseases Institute at Makerere University and the Mulago Hospital Research and Ethics Committee (IRB Study ID MHREC1246), and University of California San Francisco (IRB 13-12236).

Note that full information on the approval of the study protocol must also be provided in the manuscript.

Clinical data

Policy information about clinical studies

All manuscripts should comply with the ICMJEguidelines for publication of clinical research and a completedCONSORT checklist must be included with all submissions.

Clinical trial registration	N/A
Study protocol	N/A
Data collection	N/A
Outcomes	N/A
